# Global trends in mortality related to pulmonary embolism: an epidemiological analysis of data from the World Health Organization mortality database from 2001 to 2023

**DOI:** 10.1016/j.eclinm.2025.103389

**Published:** 2025-07-31

**Authors:** Hideharu Hagiya, Ko Harada, Yoshito Nishimura, Maki Yamamoto, Sayoko Nishimura, Michio Yamamoto, Takahiro Niimura, Yuka Osaki, Quynh Thi Vu, Mariko Fujii, Nanami Sako, Tatsuaki Takeda, Hirofumi Hamano, Yoshito Zamami, Toshihiro Koyama

**Affiliations:** a Department of Infectious Diseases, Okayama University Hospital, Okayama, 7008558, Japan; b Brookdale Department of Geriatrics and Palliative Medicine, Icahn School of Medicine at Mount Sinai, Mount Sinai Beth Israel, New York, NY, 10029, USA; c Division of Hematology and Oncology, Mayo Clinic, Rochester, MN, 55901, USA; d Department of Health Data Science, Graduate School of Medicine, Dentistry, and Pharmaceutical Sciences, Okayama University, Okayama, 7008558, Japan; e Graduate School of Human Sciences, Osaka University, Osaka, 5650871, Japan; f RIKEN Center for Advanced Intelligence Project, Tokyo, 1030027, Japan; g Department of Clinical Pharmacology and Therapeutics, Institute of Biomedical Sciences, Tokushima University Graduate School, Tokushima, 7708503, Japan; h Center for Education in Medicine and Health Sciences, Okayama University Graduate School of Medicine, Dentistry and Pharmaceutical Sciences, Okayama University, Okayama, 7008558, Japan; i Department of Pharmacy, Medical Development Field, Okayama University, Okayama, 7008558, Japan

**Keywords:** Pulmonary embolism, Mortality, WHO, Global trends

## Abstract

**Background:**

Pulmonary embolism (PE) remains a major contributor to the global disease burden. However, disparities in international trends of PE-related mortality have not been comprehensively examined across geographic, economic, and healthcare system parameters. We employed multifaceted stratification to analyse long-term trends in PE-related mortality.

**Methods:**

This epidemiological analysis used registration data from the World Health Organization Mortality Database. PE-related mortality was defined with the International Statistical Classification of Diseases and Related Health Problems, Tenth Revision codes for acute PE (I26) and any forms of venous thromboembolism (I80, I822, I828, I829, O882, O222, O223, O229, O870, O871, and O879). Countries were deemed eligible for inclusion in the analysis if they provided mortality data for 5-year age intervals up to ≥85 years, from 2001 to 2023 (last update, February 2025). Countries with incomplete age- and sex-stratified demographic data were excluded. We used locally weighted regression (LOESS) to show global trends in crude and age-standardised mortality rates. Subgroup analyses by geographic region and income level were also performed. Additionally, joinpoint regression analysis was performed to estimate the average annual per cent change (AAPC) in the age-standardised mortality trends for each country during 2010–2023.

**Findings:**

Data from 73 countries, encompassing 1,550,883 participants [57.8% (896,393) of whom were female], were eligible for the LOESS analysis, while those from 75 countries, including 915,518 participants (56.9% (520,587) of whom were female) were valid for the joinpoint analysis. The LOESS estimates of global age-standardised PE-related mortality rate (per 100,000) decreased from 3.49 (95% confidence interval [CI], 3.20–3.79) in 2001 to 2.42 (95% CI, 2.04–2.80) in 2023. The age-standardised mortality rates considerably reduced in European regions, such as Western Europe, from 5.24 (95% CI, 4.75–5.74) to 2.25 (95% CI, 1.62–2.87) in 2023; however, in Africa, they remained high from 4.23 (95% CI, 3.82–4.64) in 2001 to 3.90 (95% CI, 2.81–5.00) in 2023. High-income countries showed a continuous downward trend, from 3.68 (95% CI, 3.28–4.08) in 2001 to 2.20 (95% CI, 1.68–2.71) in 2023, whereas lower-to middle-income countries showed a rising trend, from 0.92 (95% CI, 0.04–1.81) in 2001 to 4.82 (95% CI, 3.12–6.52) in 2023. Higher increases in the age-standardised mortality rates were predominantly observed in lower-middle-income countries.

**Interpretation:**

Globally, the PE-related mortality rate has declined over the last two decades, except in countries with certain geographical and economic conditions. Despite the potential limitation of misclassification and underreporting, our efforts corroborated that greater efforts are needed to reduce PE-related mortality, especially for populations in susceptible regions and lower-middle-income countries. A multi-layered approach will increase awareness of the disease and facilitate the development of healthcare policies that enhance its clinical management.

**Funding:**

The Japan Society for the Promotion of Science, the 10.13039/100014475Pfizer Health Research Foundation, and the Ohyama Health Foundation Inc.

**Research in context**

**Evidence before this study**

We searched PubMed and Web of Science for epidemiological studies published in any language from January 1^st^ 2000 to May 1^st^ 2025, using the search terms ‘pulmonary embolism’, ‘venous thromboembolism’, ‘mortality’, ‘death’, ‘trends’, ‘international’, ‘global’, ‘national’, ‘nationwide’, and ‘World Health Organization’. Relevant studies were identified from the search results, some of which have reported time trends in pulmonary embolism (PE)-related mortality rates in a limited number of countries and regions, including Europe, USA, Canada, Australia, and Brazil. These studies showed downward and upward trends in PE-related mortality rates during the 2000s, depending on each country and time. To the best of our knowledge, the information available from these previous studies is limited to certain countries and regions, and no updated global study has yet been reported.

**Added value of this study**

This study, based on medically certified vital registration data from the WHO Mortality Database, is the first analysis of the global PE-related mortality rate in WHO member countries. The present study analyses the PE-related mortality rates in 75 countries over the past 23 years (2001–2023). According to our results, the age-standardised mortality rate related to PE has slightly but steadily decreased since 2001 until recently. Additionally, subgroup analysis showed differences in trends by region and income level.

**Implications of all the available evidence**

Despite some potential limitations, such as underreporting or misclassification of the disease, absence of patient background data, and lack of fully available data in some countries, our data corroborated that, while demonstrating a global declining trend, the PE-related deaths have remained high or even increased in low-income developing countries. This unfavourable trend calls for intensified global efforts, as PE is both preventable and treatable through a multi-faceted approach. Specifically, countries facing geographical, economic, and healthcare infrastructure disadvantages encounter significant challenges, and targeted measures should prioritise enhancing essential healthcare resources in these regions.

## Introduction

Venous thromboembolism (VTE), comprising deep vein thrombosis and pulmonary embolism (PE), is a major contributor to the global disease burden.^1^ PE is a fatal condition resulting from a venous thrombus migrating from the venous circulatory system and embolises the pulmonary artery. Its risk factors are diverse and include surgery, malignancy, ageing, and obesity,^2^ all of which are frequently encountered in modern medical settings. Notably, predispositions for PE are now more common than ever despite rapid medical advances. Previous epidemiological studies have highlighted that the incidence of PE ranges from 14 per 100,000 persons in China,^3^ to 39 per 100,000 persons per year in Hong Kong,^4^ to 115 per 100,000 persons per year in the United States.^5^

However, several nationwide studies have reported decreasing trends in PE-related mortality. For example, in Australia, the age-standardised PE-related mortality rate decreased to 1.73 per 100,000 persons in 2007.^6^ Additionally, in France, the age-standardised PE-related mortality rate decreased to 2.1 per 100,000 persons in 2010.^7^ Furthermore, Brazilian data indicated that the age-standardised mortality rate decreased from 3.04 to 2.09 per 100,000 persons between 1998 and 2010, with disparities in healthcare access and quality significantly influencing the mortality rate.^8^ In addition, an international study across Europe and North America reported a decline in the age-standardised mortality rate in most nations evaluated by the mid-2000s.^9^ Similarly, regional-level studies have corroborated the descending trend of PE-related mortality rates. Notably, the age-standardised PE-related mortality rate nearly halved from 12.8 to 6.5 per 100,000 between 2000 and 2015 in Europe.^10^ Studies have suggested that advances in diagnostic imaging have contributed to decreased PE-related fatality rates.^11^ Additionally, the development of direct oral anticoagulants has been transformative and could have a favourable influence on the mortality trend of PE.^12^^,^^13^ However, in North America, the downward trend slowed in Canada after 2006, and the mortality trend increased among young and middle-aged adults in the United States.^14^ Increased exposure to risk factors for PE and poor access to healthcare among particular populations might explain the reversal of uptrends in this region. Importantly, the global pandemic of novel coronavirus disease 2019 (COVID-19) might have increased PE-related mortality, but this has yet to be comprehensively evaluated in the literature.

PE-related mortality rates vary across countries and geographic regions as previously described, potentially due to disparities in social and population backgrounds as well as healthcare levels. Hence, recognising global mortality trends may help identify differences among countries or regions and develop global and country-specific strategies. Moreover, understanding global mortality trends allows for focused prevention efforts and prioritising the allocation of critical resources. A recent study used the World Health Organization (WHO) mortality database and presented analytical findings from the updated 2019 release, with results stratified according to countries' geographical regions and socioeconomic status classifications.^1^ However, this study lacked a well-designed trend analysis. To our knowledge, no existing literature has systematically evaluated the global PE-related mortality trends from this perspective. Therefore, in this study, we aimed to identify global trends in PE-related mortality rates by examining regional, economic, and healthcare indicators.

## Methods

### Data source

We obtained data on the number of deaths from the WHO mortality database (last update, February 2025),^15^ which is used for the routine collection of death data reported yearly by member countries from their civil registration systems. This database comprises the number of deaths reported with their underlying causes by age and sex, recorded in national vital registration systems since 1950. The database categorises age into one of nine age group formats. However, related data, such as secondary causes of death and race, are not included. Furthermore, in this database, the causes of death are coded using the International Statistical Classification of Diseases and Related Health Problems, Seventh to Tenth Revision (ICD 7–10) criteria. Deaths were considered being due to PE if the ICD-10 codes for acute PE (I26) and any forms of VTE (I80, I822, I828, I829, O882, O222, O223, O229, O870, O871, and O879) were present, following previous reports.^10^^,^^14^ From the WHO Mortality Database, countries were included if they had data available in 5-year age intervals, ranging from 0 to 4 years up to 85 years and older, from 2001 to 2023. We categorised the vital registration data quality as high, medium, or low if the latest usability from 2008 to 2019 was ≥80%, 60% to <80%, or <60%, respectively.^16^ The ‘usability’ metric, defined as the percentage of all deaths that are registered with meaningful cause-of-death information, is used to assess the overall quality of vital registration data. Countries with ‘high’ or ‘medium’ ratings for mortality data quality were included in the study. The population estimates were obtained from the United Nations World Population Prospects, 2024 Revision,^17^ which provides comprehensive demographic data for 237 countries and territories from 1950 to the present, thus serving as the foundational source for various population analyses. Mid-year population data for each nation was utilised for our analyses. Countries lacking age- and sex-specific population data were excluded, as mortality rates could not be calculated. We did not perform any imputation procedures, as the missing data were determined to be missing completely at random, as in a previous study.^18^ However, a sensitivity analysis was performed to estimate the effect of missing data on the results. Missing values were complemented using a combination of the last observation carried forward and the next observation carried backward methods, as well as linear interpolation.^19^ The results are almost consistent with those from the primary analysis (approximately 2–3% of the mean age-standardized mortality), except for the first few years of the study period, which had many missing data (Supplementary Table S1). Data processing was conducted independently by TK, QV, MF, NS, SN, and TN.

Following the United Nations ' classification, we divided the countries into seven geographic regions to identify geographic differences in PE-related mortality^20^—North America, Latin America and the Caribbean, Western Europe, Eastern Europe, Asia, Oceania, and Africa. In addition, we classified the target countries into high, upper-middle, and lower-middle income levels following the World Bank's classification, to investigate the relationship between mortality rate and economic level.^21^ We adopted the 2011 income-level data because 2011 was the median of our study period. The Healthcare Access and Quality (HAQ) index^22^ was used to explore the association between healthcare level and mortality. This index, calculated every 5 years from 1990 to 2015, is measured on a scale of 0 (worst) to 100 (best) based on 32 distinct causes of death that could be avoided by quick and appropriate management (or ‘amenable mortality’). In this study, we used Spearman's rank correlation coefficient to examine the correlation between the 2015 HAQ index and age-standardised mortality rates in 2015. For countries where the 2015 data were unavailable, we substituted the mortality rates for the nearest available year (2014 data for four countries).

### Statistical analyses

**Fig. 1:**
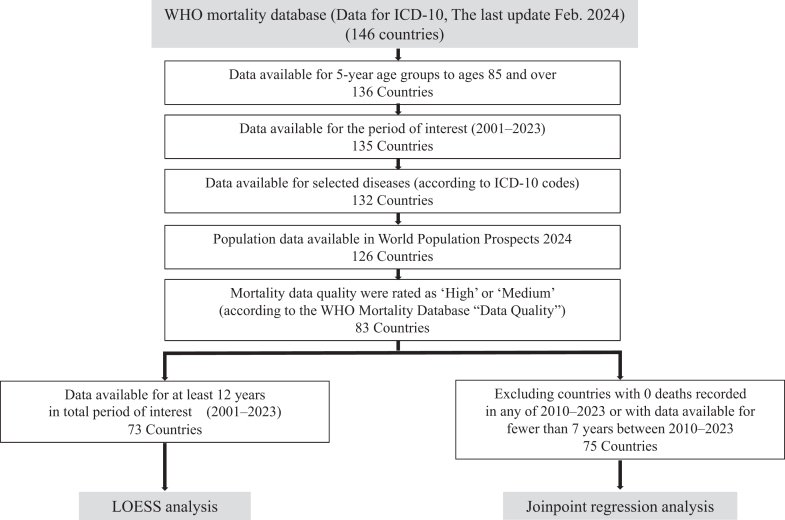
**Flow diagram of the analysis**.

The flow of statistical analysis is shown in Fig. 1. We calculated crude PE-related mortality rates per 100,000 population each year by dividing the number of PE-related deaths by the corresponding population size in each country. Furthermore, we calculated age-standardised mortality rates using the new WHO World Standard Population Distribution to improve the comparison among countries and adjust for differences in the population distribution among target countries in the study.^23^ The age-standardised mortality rates were calculated for 5-year age groups in each country using the standard population. We indicated years in which zero PE-related deaths were reported as a true zero, as previously described.^18^ The PE-related deaths were assumed to be zero for years when non-PE-related deaths and no PE-related deaths were reported. Furthermore, we used locally weighted regression (LOESS),^24^ which was weighted using population size per country, to produce a smooth curve of the global PE-related mortality rate. Countries with data available for at least 12 years of the period of interest were included in the analysis. LOESS-smoothed mortality rates, along with 95% confidence intervals (CIs), were calculated (Supplementary Table S2), and mortality rate trends were stratified by the United Nations country group and the World Bank's income groups. Furthermore, we also performed a sensitivity analysis solely including ICD-10 codes I26 and O88.2 (code O88 if sub-codes are not recorded). After excluding countries for which the number of deaths was recorded as zero in any of the years between 2010 and 2023 and those that lacked data for at least 7 years between 2010 and 2023, joinpoint regression analysis was applied to estimate the average annual per cent change (AAPC) in the age-standardised mortality trends and their 95% CIs for each country using the Joinpoint Regression Program, version 5.3.0, November 2024 (Statistical Methodology and Applications Branch, Surveillance Research Program National Cancer Institute).^25^^,^^26^ This model has the advantage of identifying the year in which significant changes in trends occur. Additionally, it can estimate the magnitude of the increase or decrease in each linear slope by calculating the annual per cent change and AAPC for the entire period. We used R version 4.4.2 (R Foundation for Statistical Computing, Vienna, Austria) for the data analysis. Data processing and aggregation were performed using Microsoft Access® 2013 (Microsoft Corporation, Redmond, WA, USA). All statistical analyses were independently conducted by TK, MY, MF, NS, and SN.

### Ethics approval

We obtained approval from the institutional review board of Okayama University Hospital (No. 2007-011). The requirement for informed consent was waived because this study was a retrospective analysis of publicly available data published by the WHO. This study was performed in accordance with the principles of the Declaration of Helsinki and followed the Reporting of Studies Conducted using Observational Routinely-collected Health Data (RECORD) Statement.

### Patient and public involvement

No individual patients were involved in the study design, outcome measures, data analysis, or interpretation of study results. No plans were made to involve patients in dissemination.

### Role of the funding source

The funder of the study had no role in study design, data collection, data analysis, data interpretation, or writing of the report. The authors had full access to the data in the study. TK and HH had final responsibility for the decision to submit for publication.

## Results

In total, 73 countries met our inclusion criteria and were included in the LOESS analysis. The total PE-associated death count in these countries was 1,550,883 which comprised 57.8% women. Approximately 73.6% (1,140,973/1,550,883) were coded as I26 (Pulmonary embolism), 23.2% (359,913 of 1,550,883) as I80 (Phlebitis and thrombophlebitis), and 3.2% as other target codes (Supplementary Table S3).

**Fig. 2:**
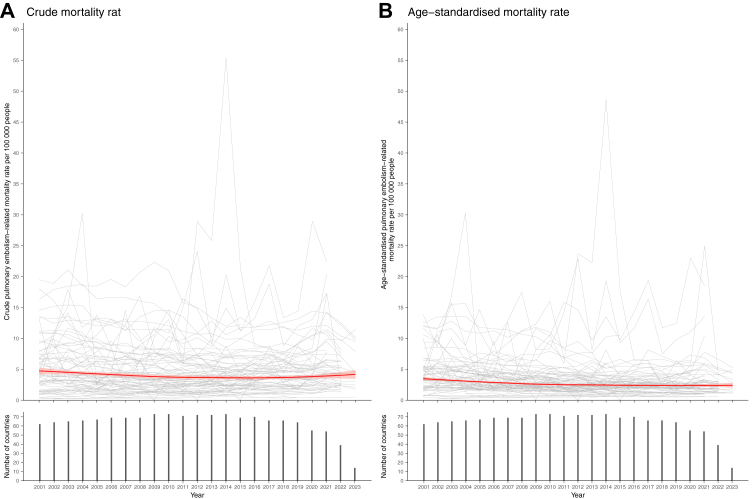
**Crude- and age-standardised pulmonary embolism-related mortality rates (per 100,000 population) in the 73 countries (2001–2023)**. The mortality rates by locally weighted regression (LOESS) analysis with 95% confidence intervals, weighted by country population, are shown in red. A. Crude mortality rate. B. Age-standardised mortality rate.

**Fig. 3:**
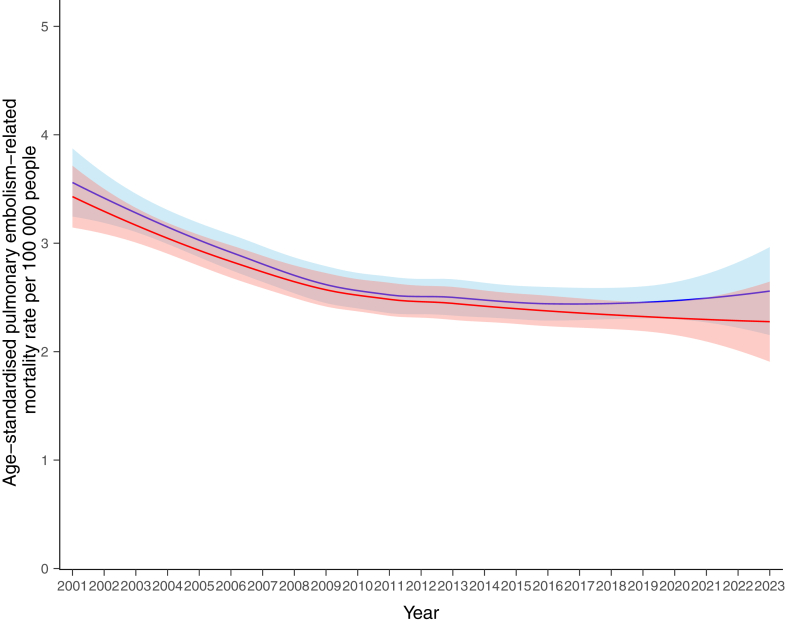
**Age-standardised pulmonary embolism-related mortality rates (per 100,000 population) by sex (2001–2023)**. The mortality rates by locally weighted regression (LOESS) analysis with 95% confidence intervals, weighted by country population, are shown in blue (men) and red (women).

The LOESS-smoothed crude and age-standardised PE-related mortality rates per 100,000 population from 2001 to 2023 are shown in Fig. 2. Detailed data on age-standardised mortality in all the included countries are given in Table 1. The LOESS-smoothed crude mortality rate started at a peak of 4.75 (95% CI, 4.20–5.30) in 2001 and followed a mild downward and flat trend (Supplementary Table S4). Stratification by age cohort (0–19 years, 20–59 years, and ≥60 years) demonstrated a favorable declining trend in the geriatric population (Supplementary Figure S1). The LOESS-smoothed age-standardised mortality rate peaked at 3.49 (95% CI, 3.20–3.79) in 2001 and declined to 2.42 (95% CI, 2.04–2.80) in 2023. A similar declining trend was observed in the sensitivity analysis for PE-specific ICD-10 codes (Supplementary Table S5). The comparison of age-standardised mortality rates between men and women for all 73 countries is provided in Fig. 3 and Supplementary Tables S6 and S7 For men, the LOESS-smoothed mortality declined from 3.56 (95% CI, 3.25–3.87) in 2001 to 2.44 in 2016–2018, and subsequently increased slightly until 2023, reaching 2.56 (95% CI, 2.15–2.96). For women, the LOESS-smoothed rate continuously dropped from 3.43 (95% CI, 3.15–3.71) in 2001 to 2.28 (95% CI, 1.91–2.65) in 2023.

**Table 1:** Age-standardised pulmonary embolism-related mortality rates for 73 countries.

Year	2001	2002	2003	2004	2005	2006	2007	2008	2009	2010	2011	2012	2013	2014	2015	2016	2017	2018	2019	2020	2021	2022	2023
LOESS smoothed rate	3.49	3.35	3.22	3.10	2.98	2.88	2.78	2.68	2.60	2.55	2.51	2.49	2.48	2.45	2.43	2.41	2.40	2.40	2.39	2.39	2.40	2.41	2.42
Upper 95% CI	3.79	3.57	3.39	3.25	3.13	3.03	2.93	2.84	2.76	2.70	2.67	2.64	2.64	2.60	2.57	2.56	2.54	2.53	2.53	2.55	2.61	2.69	2.80
Lower 95% CI	3.20	3.14	3.06	2.95	2.84	2.72	2.62	2.53	2.44	2.40	2.35	2.34	2.32	2.30	2.29	2.27	2.26	2.26	2.25	2.23	2.19	2.12	2.04
Antigua and Barbuda	11.96	11.48	15.79	8.91	14.69	8.90	12.81	17.49	8.21	8.29	14.21	22.87	8.11	19.29	9.29	11.63	16.41	7.47	9.83	11.03	13.61		
Argentina	3.40	3.26	3.15	3.06	3.03	2.90	2.98	2.71	2.69	2.81	2.97	2.81	2.92	2.94	2.98	3.14	3.10	2.71	2.57	2.54	2.96	2.92	
Armenia						4.81	3.51	3.51	4.56	5.56	5.23	4.52	2.32	3.25	2.77	3.31	3.80	4.91	5.85	7.35	6.85	4.90	
Australia	1.84	2.04	2.04	1.94		1.62	1.61	1.64	1.47	1.38	1.39	1.33	1.33	1.51	1.44	1.35	1.29	1.23	1.43	1.33	1.37	1.30	1.15
Austria		4.42	4.08	3.58	4.08	3.64	3.53	2.75	2.83	2.87	2.48	2.33	2.33	2.18	2.59	2.54	2.50	2.66	2.22	2.05	1.96	1.75	1.84
Bahamas	3.44	10.62	2.58	4.38	6.56	2.89	6.35	6.63	6.34	9.67	8.53	9.50	7.61	10.30	10.52								
Belgium	5.41	5.41	5.40	4.93	4.64	4.18	4.29	3.60	3.66	3.72	3.09	3.27	3.16	3.02	3.09	2.92	2.62	2.67	2.38	2.32	2.50		
Belize	6.38	6.47	5.15	4.48	2.49	6.38	5.04	4.63	2.13	4.75	4.37	4.50	5.51	3.85	3.48	3.64							
Brazil	5.24	5.15	5.02	4.85	4.50	4.64	4.52	4.32	4.34	4.30	4.48	4.49	4.49	4.56	4.72	4.73	4.33	4.14	4.21	3.27	3.91		
Canada	2.02	1.98	2.06	1.85	1.86	1.34	1.38	1.44	1.51	1.40	1.43	1.37	1.27	1.28	1.35	1.38	1.26	1.32	1.29	1.47	1.42	1.40	
Chile	1.67	1.77	1.68	1.90	1.72	1.56	1.64	1.47	1.81	1.84	1.38	1.25	1.65	1.85	1.77	1.93	2.01	2.01	2.32	2.10	2.42		
Colombia	4.07	4.46	4.63	4.54	5.18	4.63	4.49	4.03	3.93	3.69	3.91	3.79	3.06	2.71	2.77	2.52	2.33	2.38	2.21	2.35	3.43		
Costa Rica	5.31	3.89	3.09	3.16	2.17	2.39	2.92	2.26	2.13	2.89	2.56	2.63	2.36	2.25	1.70	2.44	2.04	2.52	3.27	2.17	2.32	1.87	
Croatia	8.44	8.50	8.57	8.26	6.76	6.38	6.73	5.28	2.98	2.08	2.49	2.12	1.74	1.78	2.00	1.84	2.20	1.62	1.19	1.05	1.18		
Cuba	3.35	2.82	2.69	2.96	2.84	2.78	2.31	2.28	2.09	2.42	2.01	2.29	2.38	2.55	2.13	2.29	3.26	3.54	4.12	4.39	10.47		
Cyprus				1.04	1.14	1.18	0.66	1.04	1.03	0.97	0.83	0.75	0.74	0.83	1.50	0.96	1.01	0.75	1.23	0.86	1.21	1.41	
Czech Republic	12.05	11.63	12.81	11.12	10.85	11.25	10.16	11.11	11.81	11.01	8.25	7.59	6.78	5.85	6.63	5.66	6.03	5.43	5.41	5.62	5.77	4.92	4.42
Denmark	3.17	3.31	3.55	3.68	2.86	2.63	2.52	2.13	2.35	2.04	2.03	1.92	1.78	1.74	1.86	1.57	1.78	1.77	1.61	1.40	1.80	1.77	
Dominica	9.95	1.86	6.71	3.34	2.16	4.15	12.66	1.91	8.12	10.55	5.95	23.72	22.22	48.68	17.57	6.39	6.40	2.47	6.44	4.04			
Ecuador	2.38	1.86	1.84	2.30	1.85	2.23	2.35	2.39	2.10	2.81	2.11	1.55	1.88	1.66	1.34	1.37	1.39	1.55	1.58	1.78	1.80	1.52	
Estonia	3.79	3.17	4.88	4.52	4.18	3.92	3.43	5.11	4.43	3.13	3.05	3.52	2.68	3.48	3.33	3.54	2.95	2.91	3.88	4.79	4.78	3.29	
Finland	3.28	3.52	3.20	3.70	3.44	3.16	3.41	2.86	2.77	3.00	2.76	2.76	2.64	2.30	2.24	2.13	2.63	1.98	2.05	2.11	1.84	1.80	
France	5.00	5.04	4.93	4.46	4.24	4.01	3.82	3.56	3.46	3.27	2.48	2.32	2.18	2.04	2.12	2.07	2.07	2.02	1.84	1.90	1.94	1.96	
Georgia	0.73			1.91	1.48	1.99	1.31		1.63	0.65	1.96	1.62	1.50	0.49	2.34	2.86	2.97	3.08	4.20	4.63	5.69		2.79
Germany	6.78	6.16	6.32	6.18	6.14	5.74	5.62	5.50	5.33	4.99	4.56	4.28	4.32	3.91	3.92	3.59	3.26	3.12	2.94	2.94	2.88	2.72	
Grenada	4.94	11.05	11.59	8.85	3.48	4.86	4.33	4.10	3.66	3.17	4.07	4.18	3.63	8.14	3.49	5.79	6.00	2.47	1.67	3.10	6.13		
Guatemala					2.17	1.79	1.76	2.09	1.88	2.33	2.12	1.98	2.71	2.47	2.09	2.28	2.17	2.30	2.18	1.85	2.93	2.99	
Guyana	1.44	3.09	4.07	3.65	3.16	4.43	3.05	3.49	0.00	4.76	3.38	4.32	3.71	5.90	4.26	4.91	5.36	4.70	2.20				
Hungary	10.62	9.46	7.21	5.93	4.05	3.58	3.82	3.54	4.04	3.74	3.20	3.49	3.19	2.90	2.68	2.06	2.15	2.49	2.12	2.46	2.91	2.52	2.10
Iceland	4.67	4.70	2.43	2.25	1.80	1.65	3.77	2.67	2.19	1.55	1.41	1.46	1.29	1.00	1.48	2.23	2.85	1.91	2.24	1.89	1.44	1.53	
Ireland							3.33	3.27	2.89	3.37	2.97	3.09	2.73	2.59	2.44	1.80	2.26	2.16	2.07	2.02	2.56	2.67	
Israel	1.88	2.42	2.14	1.94	2.08	1.93	2.05	1.92	1.84	1.60	1.47	1.56	1.60	1.78	1.47	1.32	1.26	1.24	1.21	1.17	1.43	1.19	
Italy			1.97	1.71	1.70	1.67	1.62	1.11	1.15	1.07	1.13	1.13	1.00	1.01	1.05	0.98	1.04	1.02	0.98	1.02	1.03		
Jamaica	5.56	4.72	4.30	5.42	4.68	1.70			4.02	5.14	5.29	4.86	4.11	6.09									
Japan	0.82	0.79	0.74	0.79	0.80	0.74	0.77	0.74	0.70	0.70	0.76	0.71	0.68	0.66	0.66	0.65	0.54	0.54	0.52	0.54	0.55		
Kuwait	5.02	5.43	3.58	5.90	3.02	4.07	1.65	3.81	3.23	2.56	2.72	3.97	2.80	1.83	3.36	0.77	1.20	1.35	1.01			1.12	1.21
Kyrgyzstan	3.63	3.03	3.38	2.66	2.33	2.42	2.84	2.68	1.83	2.43	1.88	1.99	2.14	2.58	2.30	2.50	2.79	2.60	3.05				
Latvia	1.73	2.38	1.90	2.14	1.70	2.49	2.28	2.44	2.24	3.49	2.48	2.26	2.55	2.39	2.31	2.85	3.45	3.59	4.13	4.85	5.03	4.12	4.95
Lithuania	5.19	5.90	5.51	5.54	6.42	7.89	7.20	5.62	6.59	5.09	5.28	5.55	6.44	5.17	5.50	5.41	5.23	5.48	5.81	6.20	5.97	5.24	4.43
Luxembourg	4.16	5.99	9.99	6.55	7.65	7.28	6.19	7.40	7.31	7.92	7.00	6.14	4.92	3.66	3.26	3.43	4.05	3.57	2.54	4.10	2.87	2.19	
Malta	4.94	3.20	3.10	2.09	2.86	2.80	2.20	4.12	1.43	1.98	1.38	2.72	2.55	1.68	2.58	1.33	1.95	2.46	2.11	2.47	3.38		
Mauritius					2.17	3.01	2.13	1.95	2.54	2.70	2.10	2.21	1.54	2.07	2.04	2.43	2.33	2.02	1.58	3.03	3.28	1.84	1.40
Mexico	2.05	2.08	2.11	2.02	1.93	2.03	1.68	1.68	1.67	1.76	1.75	1.69	1.74	1.89	2.00	2.04	1.97	2.00	1.94	1.83	2.24	2.21	
Netherlands	2.31	2.32	2.37	2.31	2.18	2.12	2.02	1.85	1.88	1.71	1.56	1.69	1.45	1.29	1.43	1.23	1.16	1.24	1.10	1.09	1.20	1.23	1.15
New Zealand	0.73	0.75	0.38	0.45	0.70	0.51	0.77	0.71	0.71	0.74	0.91	0.90	0.88	0.90	1.18	1.02	1.29	1.28					
Nicaragua	0.62	1.39	1.12	1.47	1.69	2.09	2.14	2.59	2.28	2.02	1.80	2.16	2.14	2.18	2.12	2.76	2.50	2.58	3.26	5.68	24.99	3.78	
Norway	2.10	2.37	2.28	2.59	1.64	1.76	1.77	1.89	1.56	1.63	1.57	1.61	1.32	1.51	1.69	1.37							
Panama	2.24	2.27	2.48	2.77	3.95	2.07	3.17	3.30	3.98	3.64	3.44	3.20	3.52	2.43	3.39	2.56	2.42	2.45	2.31		1.71	2.33	
Paraguay	2.37	2.57	2.97	3.07	2.68	2.18	1.74	2.19	1.99	2.01	1.77	1.48	1.24	1.02	1.16	1.52	1.40	1.10	1.32	1.36	1.89		
Philippines	0.72	0.74	0.59			0.68	0.60	0.61	0.70	0.70	0.73			1.10		0.78	0.62	0.57	0.62				
Poland	5.36	5.42	5.35	5.78	5.61	5.20	4.81	4.61	4.80	4.25	4.03	3.98	3.67	3.24	3.32	2.79	2.59	2.68	2.73	2.80	3.18	3.05	
Portugal		4.66	4.84				3.43	3.40	3.44	2.99	2.55	2.22	2.50	3.05	3.01	2.85	2.89	2.42	2.15			2.45	
Republic of Korea	0.44	0.40	0.25	0.34	0.39	0.38	0.43	0.49	0.56	0.58	0.64	0.63	0.52	0.57	0.58	0.62	0.65	0.59	0.60	0.61	0.66	0.60	
Republic of Moldova	1.48	1.67	1.44	1.88	2.28	1.92	2.56	2.07	1.98	1.70	1.77	1.91	1.92	2.29	3.06	2.05	2.71	2.73			2.56		
Romania	2.82	2.70	2.57	2.41	2.31	2.67	2.51	2.45	2.57	2.55	2.48	2.61	2.61	2.72	2.93	3.38	3.43	3.36	2.99				
Saint Kitts and Nevis	13.86	8.65	16.73	30.37	2.19	9.51	1.41	6.98	2.68	2.92	2.06	0.00	4.06	2.00	0.00	3.19							
Saint Lucia	9.40	9.41	15.53	15.63	11.12	7.22		11.26	12.28	16.02	6.85	7.62	7.76	4.10	3.63	4.45	8.14	7.10	6.17	6.46			
Saint Vincent and Grenadines	11.68	9.44	7.04	6.89	5.97	6.63	7.37	6.66	11.63	10.37	15.87	14.61	9.70	13.48	9.73	14.31	19.38	11.73	12.42	23.00	18.51		
Serbia	3.35	3.26	2.88	3.49	4.05	3.90	3.18	3.42	3.41	3.52	3.12	3.55	3.74	3.47	3.78	3.89	4.00	4.06	3.68	3.30	4.10	4.15	3.37
Slovakia	12.48	13.46	13.54	13.55	11.79	11.58	11.38	10.68	10.29	8.96		4.59	6.11	5.15		6.57	6.48	6.66	5.97	5.43	7.73	6.58	5.24
Slovenia	4.74	3.71	2.84	3.73	4.39	5.24	3.81	3.26	3.75	3.58	3.44	3.67	3.36	2.84	2.94	2.32	2.01	2.18	2.01	1.78			
South Africa	4.03	4.30	4.79	4.46	4.09	4.31	4.29	4.43	4.61	4.34	4.17	4.05	3.91	3.97	4.38	4.17	4.28	4.39	4.27	4.43			
Spain	3.33	3.32	3.26	2.84	2.99	2.64	2.72	2.65	2.45	2.05	2.01	1.95	1.94	1.90	1.99	1.85	1.84	1.86	1.79	1.99	2.20	2.18	
Suriname	3.54	6.05	5.04	1.38	3.31	4.14	2.00	1.80	2.09	3.32	1.88	3.61	3.95	3.05									
Sweden	4.21	4.35	3.92	2.55	2.57	2.41	2.33	2.34	2.39	1.92	2.35	2.46	2.22	2.03	2.02	2.00	1.92	2.10	1.90	1.83	1.97	1.78	1.82
Switzerland	3.13	3.15	3.02	2.45	2.49	2.17	2.00	2.20	2.35	2.08	2.06	2.19	1.98	2.06	2.00	2.09	1.99	1.86	1.87	2.11	1.91	1.91	
Thailand		0.22	0.30	0.35	0.41	0.40	0.36	0.40	0.42	0.43	0.48	0.52	0.59	0.72	0.73	0.71	0.75	0.84	0.83		0.95		
Turkey									2.04	2.13	2.38	2.43	2.82	3.31	2.37	2.53	2.94	2.58	2.52	2.63	3.11	2.66	2.62
United Kingdom	6.76	6.62	6.76	6.24	5.99	6.02	5.66	5.59	5.60	5.60	4.18	4.08	4.09	4.01	3.98	3.90	3.87	3.79	3.44	4.14	4.15		
United States of America	2.95	2.92	2.89	2.67	2.65	2.20	2.23	2.20	2.18	2.22	2.25	2.24	2.25	2.28	2.33	2.32	2.32	2.30	2.23	2.52	2.47	2.27	
Uruguay	4.85	5.17	5.23	5.50	5.40	4.53	4.26	4.01	3.84	4.18		3.74	2.74	2.09	2.50	2.89	2.79	2.98	2.84	2.63	3.27	2.99	
Uzbekistan				1.17	0.55				1.53	1.56	1.15	1.30	1.94	2.72	4.62	4.45	4.88	5.12	4.53				
Venezuela	1.62	1.51	1.58	1.74	1.61	1.89	1.93	1.82	2.08	2.20	1.84	1.72	1.68	2.03	2.09	3.24							

Data are number of deaths per 100,000 people. LOESS, locally weighted regression; CI, confidence interval.

According to the 2025 database update, mortality counts fewer than three for specific age groups are represented as missing values for Germany from 2021 onward.

**Fig. 4:**
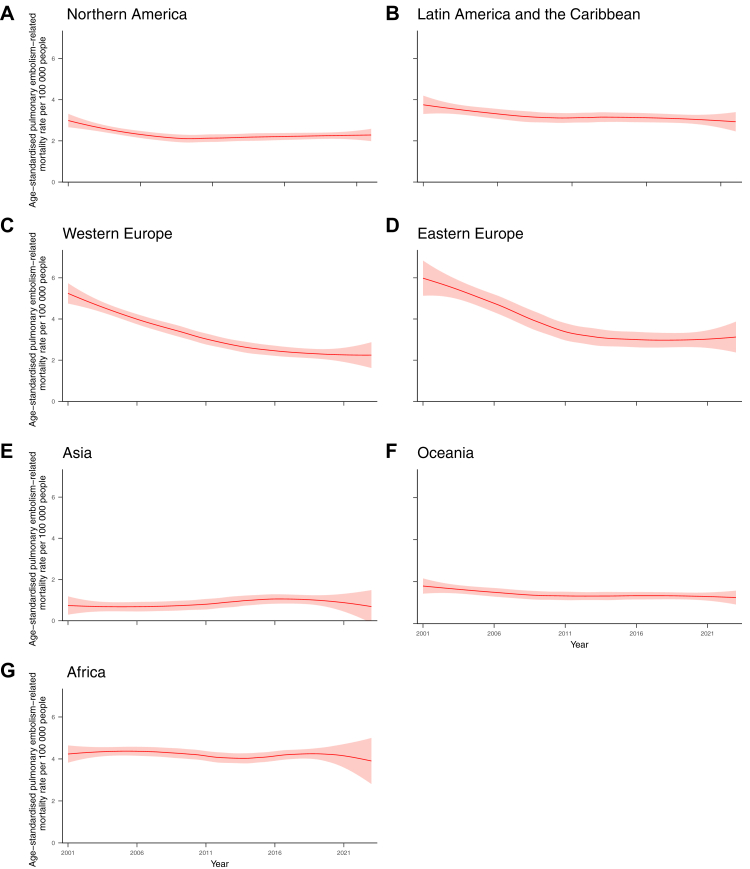
**Age-standardised pulmonary embolism-related mortality rates (per 100,000 population) by geographic region (2001–2023)**. The mortality rates by locally weighted regression (LOESS) with 95% confidence intervals, weighted by country population, are shown in red for A. Northern America, B. Latin America and the Caribbean, C. Western Europe, D. Eastern Europe, E. Asia, F. Oceania, G. Africa. A breakdown list of countries included in the LOESS analysis is provided in Supplementary Table S9.

The geographical comparisons of age-standardised PE-related mortality rates for the 73 countries are shown in Fig. 4 and presented in detail in Supplementary Tables S8 and S9 North America showed a decrease from 2.99 (95% CI, 2.66–3.31) in 2001 to 2.11 (95% CI, 1.92–2.30) in 2009; however, the trend mildly increased in the latter half of the reporting period, reaching 2.28 (95% CI, 1.99–2.58) in 2022. Latin America and the Caribbean showed a slightly declining and levelling-off trend from 3.75 (95% CI, 3.30–4.20) in 2001 to 2.93 (95% CI, 2.46–3.39) in 2022. Both Western and Eastern Europe recorded the highest mortality rates in 2001, at 5.24 (95% CI, 4.75–5.74) and 5.98 (95% CI, 5.13–6.84), respectively. The mortality rate in Western Europe continuously declined over time, reaching 2.25 (95% CI, 1.62–2.87) in 2023. In contrast, Eastern Europe exhibited a downward trend until 2018, with the lowest at 2.97 (95% CI, 2.62–3.32), after which it began to rise again. We observed a flat trend in Asia, approximately 0.7 in the first half, followed by a slow upward and downward trend thereafter. The mortality rate in Oceania decreased slowly from 1.79 (95% CI, 1.43–2.15) in 2001 to 1.24 (95% CI, 0.91–1.57) in 2023. Africa began with a high mortality rate at 4.23 (95% CI, 3.82–4.64) in 2001, which remained at the same level for more than a decade, and finally dropped to 3.90 (95% CI, 2.81–5.00) in 2023.

**Fig. 5:**
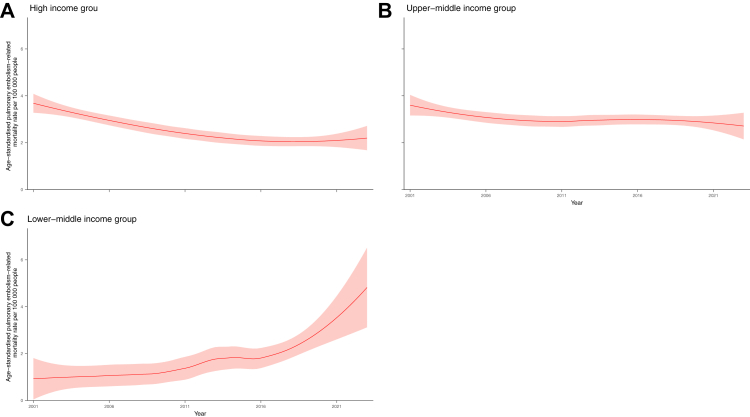
**Age-standardised pulmonary embolism-related mortality rates (per 100,000 population) by income level (2001–2023)**. The mortality rates by locally weighted regression (LOESS) with 95% confidence intervals, weighted by country population, are shown in red for A. High income group, B Upper-middle income group, C. Lower-middle income group. A breakdown list of countries included in the LOESS analysis is provided in Supplementary Table S11.

We divided the eligible countries into high, upper-middle, and lower-middle income groups to compare age-standardised mortality rates by income level (Fig. 5, Supplementary Tables S10 and S11). The high-income group showed a continuously declining trend during the study period, from 3.68 (95% CI, 3.28–4.08) in 2001 to 2.20 (95% CI, 1.68–2.71) in 2023. The upper-middle income group showed a mild decrease from 3.60 (95% CI, 3.16–4.04) in 2001 to 2.71 (95% CI, 2.14–3.28) in 2023. However, the mortality rate in the lower-middle group upsurged from 0.92 (95% CI, 0.04–1.81) in 2001 to 4.82 (95% CI, 3.12–6.52) in 2023. Subsequently, the correlation between the HAQ index and age-standardised mortality rates was investigated, and the correlation coefficient observed was −0.37 (95% CI, −0.55 to −0.15), indicating a weak negative correlation (Supplementary Figure S2).

**Fig. 6:**
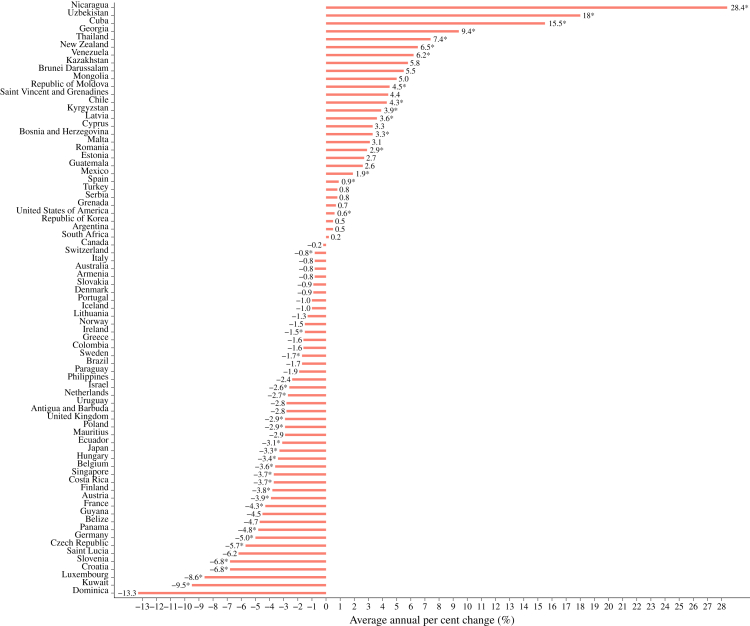
**Average annual per cent changes of age-standardised pulmonary embolism-related mortality rates (per 100,000 population) by country (2010–2023)**. ∗Indicates that average annual per cent changes (AAPCs) were statistically significantly different from zero.

Data for 75 countries were eligible for the joinpoint trend analysis. AAPC of age-standardised PE-related mortality rates for each country is shown in Fig. 6 and presented in detail in Supplementary Table S12. The highest increases were observed in Nicaragua (AAPC: 28.4% [95% CI, 16.8–65.8]), followed by Uzbekistan (AAPC: 18.0% [95% CI, 12.9–28.1]), Cuba (AAPC: 15.5% [95% CI, 11.6–19.9]), and Georgia (9.4% [95% CI, 4.0–22.7]). In contrast, the lowest decrease was observed in Dominica (AAPC: −13.3% [95% CI, −41.2 to 13.2]), followed by Kuwait (AAPC: −9.5% [95% CI, −16.0 to −3.7]), Luxembourg (AAPC: −8.6% [95% CI, −11.6 to −5.5]), Slovenia (AAPC: −6.8% [95% CI, −8.3 to −5.6]), and Croatia (AAPC: −6.8% [95% CI, −12.2 to −2.4]).

## Discussion

This study's findings reveal a temporal analysis of the trends in PE-related mortality rates over 23 years across 73 countries, utilising information from the WHO Mortality Database. Overall, the age-standardised mortality rate has gradually declined since 2001 for both males and females. Geographically, the age-standardised mortality rates have significantly decreased in European countries; however, the decrease was not observed in other regions. Furthermore, the countries in the high-income group demonstrated a continuous declining trend, whereas lower-middle-income countries exhibited a notable increase, especially in the last decade. Lower healthcare quality was weakly correlated with increased age-standardised mortality rates.

The observed overall downward trend in age-standardised PE-related mortality rate aligns with reports from preceding national-level studies.^6^, ^7^, ^8^^,^^10^ This improvement could be partially attributed to significant advances in PE management. Recent reviews have revealed substantial progress in diagnostic approaches.^12^^,^^27^ The development and enhancement of clinical decision rules—such as the pulmonary embolism rule-out criteria, Wells score, revised Geneva score, simplified Geneva score, and YEARS items^28^—have contributed to the early diagnosis and exclusion of PE. Notably, the D-dimer is a sensitive laboratory indicator for PE with 98%–99% sensitivity and 37%–40% specificity,^29^ which has long been used in various clinical settings. However, it can result in false positives, particularly among older populations and hospitalised patients; therefore, age-adjusted D-dimer testing has been proposed recently.^30^ Furthermore, significant progress in imaging equipment—notably ultrasonography, enhanced computed tomography, and ventilation-perfusion lung scanning—has facilitated the early detection of PE, although the clinical application of these diagnostic approaches differs worldwide. Notably, 1.2% of patients with a negative enhanced computed tomography result developed PE within 3 months, indicating that a negative finding does not entirely rule out the possibility of PE.^29^ Despite the absence of direct evidence, enhanced accessibility to computed tomography scanning might also have reduced the underdiagnosis of PE. Diagnostic algorithms for PE with high sensitivity and specificity have been established by integrating these technologies.^12^ Advances in the therapeutic field may have also reduced PE-related mortality rates, specifically the introduction of direct oral anticoagulants.^31^^,^^32^ These medicines act rapidly, thereby decreasing the risk of bleeding, eliminating the need for laboratory monitoring and fine dose adjustments, and substantially simplifying the treatment strategies for PE with increased effectiveness.^33^ Clinical guidelines for PE have been implemented in several countries and regions by consolidating existing evidence regarding these advancements.^34^^,^^35^ The development of these evidence-based support resources may also have contributed to reducing the mortality rate related to PE globally.

However, the decreasing trend in the overall mortality rate has slowed and ceased declining in the latter years of our research period. This tendency has previously been observed in a study from North America,^14^ which may be attributed to various challenges faced in modern clinical settings. First, the global increase in obesity—a significant risk factor for VTE^36^ is noteworthy. The prevalence of overweight and obesity has increased substantially in the last few decades, posing a significant health challenge worldwide.^37^ In 2013, the proportions of adult men and women with a body-mass index ≥25 kg/m^2^ were 36.9% (95% CI, 36.3–37.4) and 38.0% (95% CI, 37.5–38.5), respectively. However, in developed countries, the rise of adult obesity rates has reportedly slowed since 2006,^37^ possibly explaining the continuous decline in age-standardised PE-related mortality rate among high-income countries. Second, the growing incidence of cancer worldwide should be considered. As of 2017, the world has witnessed 24.5 million malignant cases, a 33% increase compared with that observed in 2007.^38^ During this decade, developed countries reportedly had the lowest increase in malignancy incidence,^38^ which could explain the decrease and plateau of the age-standardised PE-related mortality rate in the European and North American regions.^39^ Moreover, a global escalation in population ageing^40^ and increased surgical procedures,^41^ are both risk factors for PE,^42^^,^^43^ and may also have influenced our results.

Variations in the quality and accessibility of essential care can predominantly explain geographic differences in age-standardised mortality trends. Our results indicate that the African region consistently showed the highest PE-related mortality rate during the study period. The more severe the clinical condition, the more crucial it is to provide rapid and multidisciplinary treatment for patients with PE. Thus, poor access to healthcare services and the unavailability of high-quality medical care in these regions seem to be the main factors.^8^ Stratification data by income level corroborated that the age-standardised PE-related mortality rate in high-income countries has consistently decreased, whereas those in lower-middle-income countries have surged during these years. This can be explained by the fact that there was a potential association between a lower HAQ index and higher age-standardised mortality rates. Owing to underdiagnosis, PE-related mortality was likely underestimated during the early study period, especially in lower-to middle-income countries. The absence of granular data from those countries precludes a detailed analysis of this potential bias. Racial differences may also contribute to the rise in PE-related mortality rates in certain countries or regions. For example, a previous study using data from the United States has reported that PE-related mortality rates may vary by race; African Americans had a 50% higher mortality rate compared to white individuals, while Caucasian individuals had a 50% higher mortality rate compared to individuals from other racial groups.^14^ Thus, it is essential to intensify efforts to enhance the management of patients with PE in these countries.

The strengths of this study include the use of the WHO Mortality Database to identify trends in international PE-related mortality rates across 73 countries from 2001 to 2023, highlighting specific differences in trends according to sex, regional, and economic factors. Furthermore, we examined trends in PE-related mortality rates for each country from 2010 to 2023. However, concerns regarding the nature of the data source and methodology should be considered when interpreting our findings. First, the WHO Mortality Database compiles data on underlying causes of death from member states; hence, misclassification may have resulted in under-reporting PE as a primary cause of death. Changes in cause-of-death classification methodologies and the transition to the updated ICD-10 version may influence the accuracy and reliability of reported PE mortality data. Deaths due to PE may not have been appropriately diagnosed and classified because patients with PE present with non-specific symptoms; studies comparing clinical diagnoses and autopsy findings have shown that PE is easily overlooked in antemortem clinical diagnoses.^44^ Moreover, mortality classification codes may be more strongly influenced by reimbursement policies, which vary considerably across countries and chronologically, than clinical findings and pathophysiological diagnoses. Second, the WHO Mortality Database does not include information on race, comorbidities, or social status, precluding the possibility of focusing on patient subgroups. Third, estimated mortality results were limited to 73 countries with available data. Moreover, not all 73 countries had full-available data for the period of interest, especially countries in the lower-to middle-income group. Therefore, we limited countries with high and medium data quality and available data for at least 12 years to improve the robustness and accuracy of the study results. Fourth, this study used age-standardised mortality rates based on the new WHO World Standard Population Distribution to evaluate countries with different age structures over time. Therefore, discrepancies may exist between the findings of this study and the actual crude mortality rates of the respective countries. Fifth, data from approximately half of the lower-to middle-income countries were missing for some or several of the study years, potentially compromising the reliability of the results. Sixth, although the global COVID-19 pandemic has reportedly increased PE-related mortality,^45^ we could not comprehensively assess this impact because of the diminished number of countries included in the analysis after 2020. Further evaluation incorporating subsequently deposited data encompassing a more extensive cohort of target countries will be essential to characterise its influence comprehensively. Seventh, due to the nature of a dataset that only comprises the number of deaths, we could not calculate the case fatality rate for PE, which is defined as the proportion of diagnosed cases resulting in mortality. The case fatality rate represents an equally important metric for evaluating the clinical burden of PE from a public health perspective, as it directly measures disease severity. Elucidation of its global trends requires datasets containing detailed information on individual cases. Finally, we used the LOESS method to assess international trends. This method is robust to outliers; however, the CI may occasionally be less than zero. Therefore, caution is required when comparing mortality rates across countries because of these limitations. These facts suggest that our approach provides an incomplete picture of global PE-related mortality. Nonetheless, our findings clearly illustrate the trends in PE-related mortality rates worldwide from various perspectives, providing important insights for improving the prognosis of patients with PE.

In conclusion, we analysed PE-related mortality rates using the WHO Mortality Database and found that the global PE-related mortality rate steadily declined until recently. However, this downward trend was not universally observed, varying by geographic and economic factors. The observed trends may not accurately reflect real-world incidences due to potential underreporting and incomplete data capture. Nevertheless, PE remains a global challenge in this era of global ageing, rising obesity rates, and more invasive medical interventions. Hence, continuous efforts are required to improve clinical management and closely analyse mortality trends in large-scale epidemiological studies.

## Contributors

Conceptualization, TK and HH; methodology, TK, QV, SN, YO, MY, MF and NS; access and verification of the underlying study data, HH, TK, QV, NS and MF; validation, MY, QV, MF, TN, NS and TK; formal analysis, TK, SN, QV, MF and NS; investigation, HH, QV, MF, NS and TK; resources, HH and TK; data curation, HH, TK; writing—original draft preparation, HH; writing—review and editing, KH, YN, MY, TT, HH, YZ, TK; supervision, HH and TK; project administration, HH and TK; funding acquisition, HH and TK. All authors had full access to all data, interpreted the results, and critiqued the manuscript. All authors gave final approval to the submitted manuscript. HH and TK is responsible for the overall content as guarantor. The corresponding author attests that all listed authors meet the authorship criteria and that no others meeting the criteria have been omitted.

## Data sharing statement

The datasets generated and analysed during the current study are available from the corresponding author upon reasonable request after publication.

## Declaration of interests

The authors declare that they have no conflicts of interest.

## Appendix A Supplementary data

Abstract in Japanese
