# Supplementary material for: Global trends in mortality related to pulmonary embolism: an epidemiological analysis of data from the World Health Organization mortality database from 2001 to 2023
Source: eClinicalMedicine. 2025 Jul 31;86:103389. doi: 10.1016/j.eclinm.2025.103389 (PMC12336653; doi:10.1016/j.eclinm.2025.103389)
Supplement: Abstract in Japanese [file mmc1.docx]

***The following translations in Japanese were submitted by the authors and we reproduce them as supplied. They have not been peer reviewed. Our editorial processes have only been applied to the original abstract in English, which should serve as reference for this manuscript.***

**背景**

肺血栓塞栓症(PE, Pulmonary Embolism)は、世界的な疾病負荷の主要な要因であり続けているが、PE関連死亡率の国際的動向における格差については包括的に検討されていない。我々は、PE関連死亡率の長期動向を解析するために多面的層別化解析を実施した。

**方法**

本解析では、世界保健機関の死亡率データベースを使用した。PE関連死亡率は、急性PE（I26）およびあらゆる形態の静脈血栓塞栓症（I80、I822、I828、I829、O882、O222、O223、O229、O870、O871、O879）に対する国際統計分類第10版コードで定義した。2001年から2023年（最終更新：2025年2月）まで5歳間隔の死亡率データを提供している国を解析対象とした。人口統計データが不完全な国は除外した。粗死亡率および年齢標準化死亡率の世界的動向を示すために、局所重み付け回帰関数（LOESS）を使用した。地理および所得水準による層別化解析も実施した。さらに、2010年から2023年における各国の年齢標準化死亡率動向の年平均変化率（AAPC）を推定するために、Joinpoint解析を実施した。

**結果**

73か国からのデータが得られ、1,550,883名の参加者［うち57.8%（896,393）が女性］がLOESS解析に適格であった一方、75か国からのデータを含む915,518名の参加者（うち56.9%（520,587）が女性）がJoinpoint解析の対象となった。世界の年齢標準化PE関連死亡率（人口10万人当たり）のLOESS推定値は、2001年の3.49（95%信頼区間［CI］、3.20～3.79）から2023年には2.42（95%CI、2.04～2.80）へと減少した。年齢標準化死亡率は、欧州地域では2001年の5.24（95%CI、4.75～5.74）から2023年の2.25（95%CI、1.62～2.87）へと著しく減少した。しかし、アフリカでは2001年の4.23（95%CI、3.82～4.64）から2023年の3.90（95%CI、2.81～5.00）と高値を維持した。高所得国では2001年の3.68（95%CI、3.28～4.08）から2023年には2.20（95%CI、1.68～2.71）へと継続的な下降傾向を示した一方、低・中所得国では、2001年の0.92（95%CI、0.04～1.81）から2023年の4.82（95%CI、3.12～6.52）へと上昇傾向を示した。年齢標準化死亡率は、主に低中所得国において大きく上昇した。

**結語**

世界的なPE関連死亡率は過去20年間で減少してきたが、特定の地理的および経済的条件を有する国々では必ずしも減少トレンドは観察されなかった。我々の解析アプローチには誤分類および過少報告の可能性を含んでいるが、世界的なPE関連死亡率をさらに削減するためのより大きな努力が必要であることが示唆された。多面的疫学解析アプローチは、PEの疾病負荷を軽減するための医療政策の策定に有用だろう。
