# Supplementary material for: Global trends in mortality related to pulmonary embolism: an epidemiological analysis of data from the World Health Organization mortality database from 2001 to 2023
Source: eClinicalMedicine. 2025 Jul 31;86:103389. doi: 10.1016/j.eclinm.2025.103389 (PMC12336653; doi:10.1016/j.eclinm.2025.103389)
Supplement: Supplementary Figure [file mmc2.pdf]

The List of supplementary materials

Supplementary figures

Supplementary Figure 1. Crude pulmonary embolism-related morality rates for 73 countries by age

Supplementary Figure 2. Correlation between healthcare access, quality index, and age-standardized pulmonary embolism-related mortality rate (per 100,000 population). Spearman's rank correlation coefficient was calculated.

Supplementary tables

Supplementary Table 1. Sensitivity analysis - estimation of missing years

Supplementary Table2. Calculation of confidence intervals for the locally weighted regression and the average annual percent change

Supplementary Table 3. Details of pulmonary embolism-related mortality according to ICD-10 codes included in the study for 73 countries

Supplementary Table 4. Crude pulmonary embolism-related mortality rates for 73 countries

Supplementary Table 5. Age-standardised PE-related mortality rates in sensitivity analysis

Supplementary Table 6. Age-standardised pulmonary embolism-related mortality rates for 73 countries for male

Supplementary Table 7. Age-standardised pulmonary embolism-related mortality rates for 73 countries for female

Supplementary Table 8. Age-standardised pulmonary embolism-related mortality rates for 73 countries by geography

Supplementary Table 9. Geographical breakdown list of countries included in LOESS analysis

Supplementary Table 10. Age-standardised pulmonary embolism-related mortality rates for 73 countries by income level

Supplementary Table 11. Income level breakdown list of countries included in LOESS analysis

Supplementary Table 12. Average annual per cent changes in age-standardised pulmonary embolism-related mortality rate.

Supplementary Figure 1. Crude pulmonary embolism-related morality rates for 73 countries by age

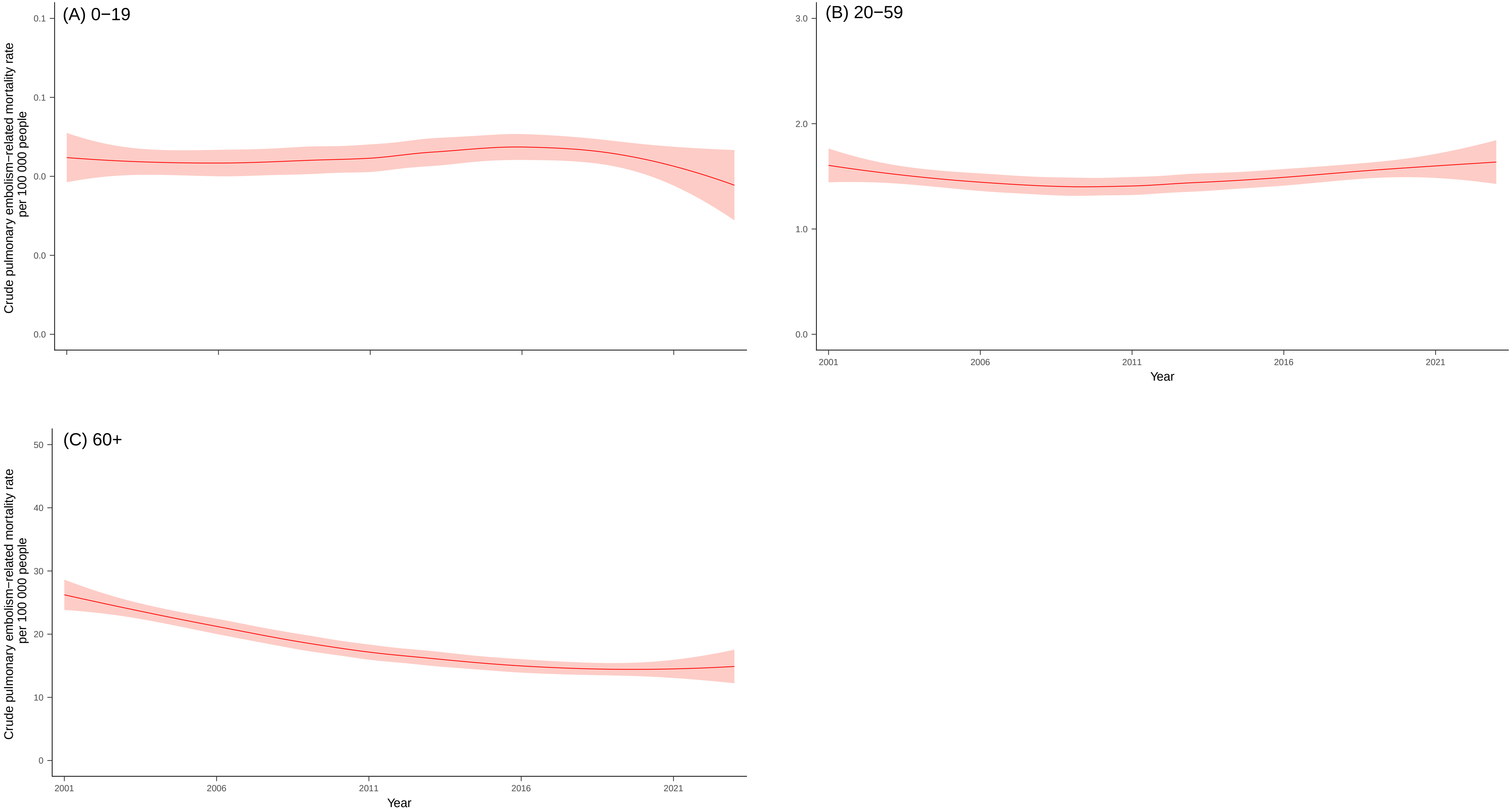

Supplementary Figure 2. Correlation between healthcare access, quality index, and age-standardized pulmonary embolism-related mortality rate (per 100,000 population). Spearman's rank correlation coefficient was calculated.

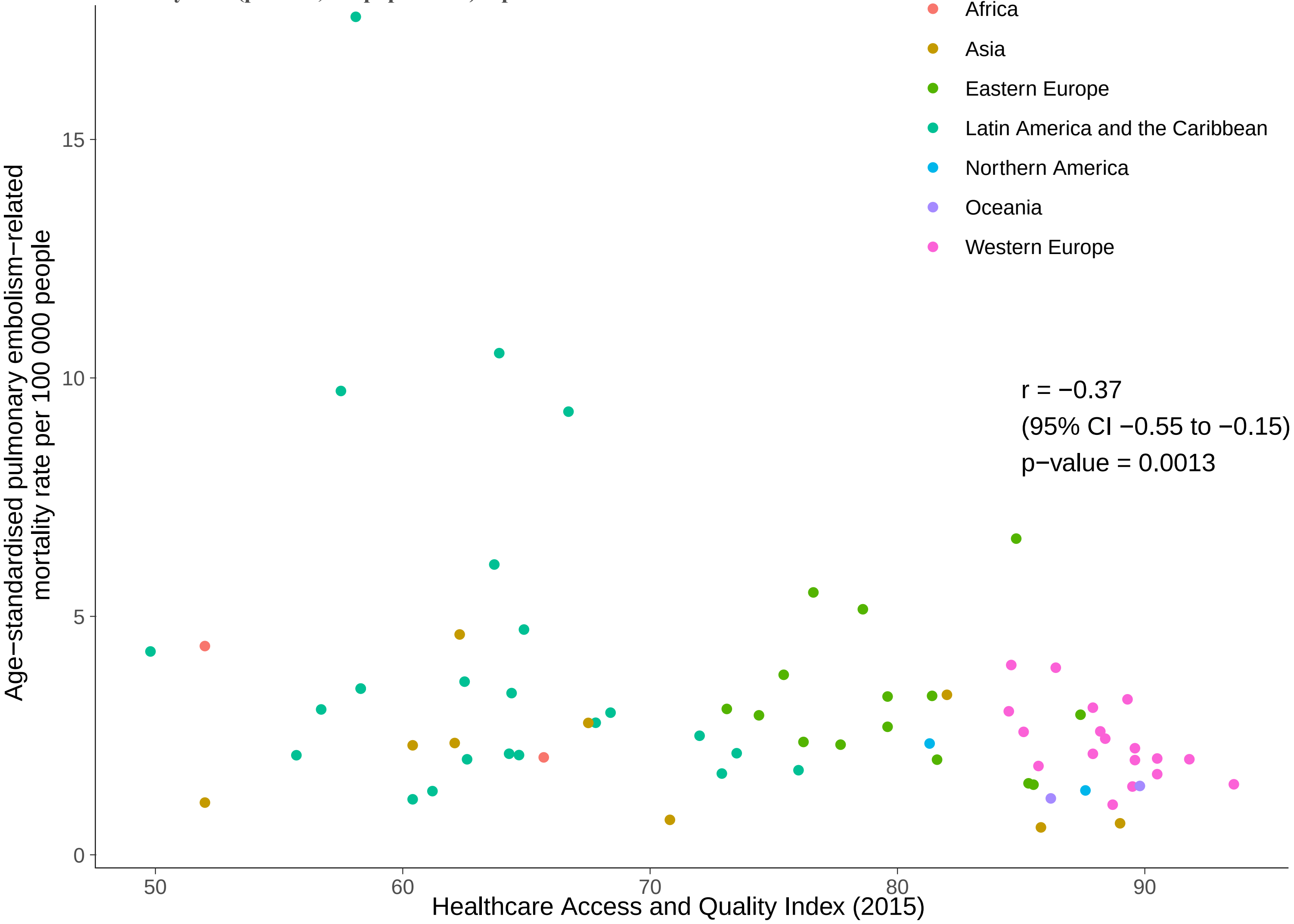

Supplementary Table 1. Sensitivity analysis - estimation of missing years

Year	Primary analysis	Linear interpolation		Last value carried forward and next observation carried backward	
		Value	Difference(%)	Value	Difference(%)
2001	3.49	3.29	-5.87	3.29	-5.85
2002	3.35	3.18	-5.24	3.18	-5.24
2003	3.22	3.08	-4.60	3.07	-4.62
2004	3.10	2.98	-3.94	2.98	-3.98
2005	2.98	2.89	-3.28	2.88	-3.34
2006	2.88	2.80	-2.65	2.80	-2.73
2007	2.78	2.72	-2.01	2.72	-2.11
2008	2.68	2.64	-1.37	2.64	-1.49
2009	2.60	2.58	-0.89	2.57	-1.03
2010	2.55	2.53	-0.75	2.53	-0.88
2011	2.51	2.50	-0.62	2.49	-0.74
2012	2.49	2.46	-1.08	2.46	-1.18
2013	2.48	2.44	-1.67	2.44	-1.74
2014	2.45	2.42	-1.42	2.42	-1.47
2015	2.43	2.40	-1.11	2.40	-1.14
2016	2.41	2.39	-0.87	2.39	-0.89
2017	2.40	2.39	-0.66	2.39	-0.67
2018	2.40	2.39	-0.42	2.39	-0.42
2019	2.39	2.39	-0.14	2.39	-0.14
2020	2.39	2.40	0.17	2.40	0.18
2021	2.40	2.41	0.51	2.41	0.52
2022	2.41	2.43	0.87	2.43	0.89
2023	2.42	2.45	1.26	2.45	1.27

Supplementary Table2. Calculation of confidence intervals for the locally weighted regression and the average annual percent change

(1) Calculation of confidence intervals for LOESS analysis

Confidence intervals (CI) of locally weighted regression (LOESS) calculated using the following formula:

$CI=EV \pm t \times SE$

EV: estimated value (e.g., estimated rate)

SE: the standard error of the estimate

t: critical value from the t-distribution with degrees of freedom (df) determined according to the "look-up degrees of freedom" approach proposed by Cleveland, et al. (1992)[1], and desired confidence level (95%)

Reference

1. W. S. Cleveland, E. Grosse and W. M. Shyu (1992) Local regression models. Chapter 8 of *Statistical Models in S* eds J.M. Chambers and T.J. Hastie, Wadsworth & Brooks/Cole.

(2) Calculation of confidence intervals for AAPC

The confidence interval for the average annual percent change (AAPC) is calculated using the empirical quantile method[2,3] implemented in the Joinpoint Regression Program version 4.2 and later. This method was introduced to address the conservative nature of the asymptotic confidence interval. The procedure involves generating resampled data by first sampling residuals from the empirical distribution of the original model residuals. This is done by transforming uniform random variables (between 0 and 1) through the inverse of the empirical distribution function of the residuals. The resampled residuals are then added to the fitted values of the model to create new datasets. For each of these datasets, the model is refitted, and the AAPC is re-estimated. The confidence interval is then obtained by calculating the $100(\alpha/2)$th and $100(1-\alpha/2)$th percentiles of the resampled AAPC estimates, corresponding to the lower and upper bounds of the $100(1-\alpha)$ % confidence interval.

References

2. National Cancer Institute, Surveillance Research Program. A description of empirical quantile confidence interval, https://surveillance.cancer.gov/help/joinpoint/setting-parameters/method-and-parameters-tab/apc-aapc-tau-confidence-intervals/empirical-quantile, [accessed July 30, 2024].

3. Kim HJ, Luo J, Chen HS, Green D, Buckman D, Byrne J, et al. Improved confidence interval for average annual percent change in trend analysis. Stat Med. 2017;36:3059–74. https://doi.org/10.1002/sim.7344.

Supplementary Table 3. Details of pulmonary embolism-related mortality according to ICD-10 codes included in the study for 73 countries

ICD-10 Codes	Description	Percentage(%)	(number of death)
Acute pulmonary embolism			
I26x	Pulmonary embolism	73.57	(1140973/1550883)
Any forms of venous thromboembolism			
I80x	Phlebitis and thrombophlebitis	23.21	(359913/1550883)
I82	Other venous embolism and thrombosis (used if sub-codes not recorded)	0.22	(3377/1550883)
I822	Embolism and thrombosis of vena cava	0.25	(3950/1550883)
I828	Embolism and thrombosis of other specified veins	0.50	(7764/1550883)
I829	Embolism and thrombosis of unspecified vein	1.91	(29565/1550883)
O222	Superficial thrombophlebitis in pregnancy	<0.01	(20/1550883)
O223	Deep phlebothrombosis in pregnancy	0.02	(294/1550883)
O229	Venous complication in pregnancy, unspecified	0.01	(149/1550883)
O870	Superficial thrombophlebitis in the puerperium	<0.01	(6/1550883)
O871	Deep phlebothrombosis in the puerperium	0.02	(233/1550883)
O879	Venous complication in the puerperium, unspecified	0.01	(152/1550883)
O882	Obstetric blood-clot embolism (code O88 if sub-codes not recorded)	0.29	(4487/1550883)

Supplementary Table 4. Crude pulmonary embolism-related mortality rates for 73 countries

Year	2001	2002	2003	2004	2005	2006	2007	2008	2009	2010	2011	2012	2013	2014	2015	2016	2017	2018	2019	2020	2021	2022	2023
LOESS smoothed rate	4.75	4.63	4.51	4.39	4.27	4.16	4.04	3.93	3.83	3.76	3.71	3.68	3.67	3.64	3.62	3.62	3.64	3.69	3.75	3.83	3.92	4.04	4.18
Upper 95% CI	5.30	5.03	4.82	4.66	4.55	4.45	4.33	4.22	4.13	4.04	4.00	3.97	3.96	3.92	3.89	3.89	3.90	3.94	4.01	4.13	4.32	4.57	4.89
Lower 95% CI	4.20	4.23	4.20	4.11	4.00	3.87	3.76	3.64	3.53	3.47	3.41	3.40	3.37	3.36	3.35	3.35	3.38	3.43	3.49	3.53	3.53	3.51	3.46
Antigua and Barbuda	9.20	10.39	14.12	8.89	13.82	8.68	12.24	16.89	8.32	8.20	15.06	24.07	9.08	20.28	11.18	13.34	18.79	8.80	12.04	13.06	17.33		
Argentina	3.95	3.87	3.76	3.68	3.69	3.65	3.77	3.44	3.40	3.66	3.86	3.70	3.88	3.95	4.10	4.31	4.29	3.80	3.68	3.61	4.27	4.27	
Armenia						5.38	4.27	4.32	5.87	7.34	7.15	6.16	3.18	4.65	4.01	4.93	6.00	7.56	9.23	11.59	10.59	7.50	
Australia	2.92	3.29	3.18	3.12		2.54	2.53	2.67	2.36	2.22	2.26	2.19	2.22	2.46	2.38	2.24	2.24	2.12	2.42	2.36	2.43	2.33	2.13
Austria		8.80	8.25	7.43	8.64	8.05	7.61	6.24	6.33	6.46	5.64	5.54	5.46	5.22	6.00	5.81	5.99	6.45	5.12	4.99	4.65	4.24	4.44
Bahamas	3.36	9.95	2.38	4.41	6.40	2.87	6.51	6.70	6.62	10.34	9.15	10.12	8.71	11.77	12.20								
Belgium	11.14	11.30	11.19	10.41	9.87	8.79	9.29	8.10	8.28	8.42	7.19	7.29	7.32	6.79	7.10	6.88	6.20	6.24	5.75	5.45	5.79		
Belize	2.82	3.52	2.65	2.58	1.43	3.47	2.70	2.30	1.28	2.50	2.75	2.69	3.22	2.29	1.96	2.19							
Brazil	3.21	3.21	3.23	3.20	3.08	3.25	3.30	3.29	3.43	3.52	3.82	3.98	4.12	4.34	4.65	4.81	4.54	4.48	4.69	3.72	4.54		
Canada	3.03	3.10	3.20	2.90	3.01	2.22	2.31	2.39	2.55	2.41	2.49	2.42	2.27	2.35	2.45	2.54	2.34	2.43	2.39	2.63	2.67	2.70	
Chile	1.57	1.72	1.69	1.98	1.87	1.76	1.89	1.75	2.25	2.32	1.73	1.66	2.22	2.55	2.50	2.83	2.94	2.95	3.47	3.25	3.80		
Colombia	2.44	2.69	2.83	2.83	3.31	3.04	3.05	2.85	2.87	2.78	3.03	3.01	2.55	2.33	2.45	2.29	2.16	2.27	2.16	2.35	3.47		
Costa Rica	3.82	2.90	2.27	2.50	1.76	2.01	2.53	2.03	1.98	2.79	2.54	2.68	2.46	2.43	1.91	2.81	2.44	3.03	4.08	2.84	3.06	2.54	
Croatia	14.53	15.19	15.42	15.31	12.73	12.16	13.18	10.11	5.96	4.28	5.14	4.23	3.63	3.89	4.47	4.06	5.03	3.48	2.96	2.45	2.78		
Cuba	4.33	3.78	3.77	4.20	4.16	4.11	3.48	3.54	3.31	3.89	3.38	3.79	4.06	4.45	3.70	4.02	5.96	6.48	7.62	8.45	20.49		
Cyprus				1.28	1.45	1.61	0.93	1.37	1.44	1.33	1.22	1.12	1.10	1.25	2.13	1.62	1.51	1.26	2.10	1.54	1.97	2.40	
Czech Republic	19.50	18.90	21.11	18.51	18.48	19.55	18.32	20.67	22.34	21.00	15.81	15.02	13.31	11.95	13.40	11.79	12.79	11.73	11.35	11.95	12.08	10.36	9.50
Denmark	5.71	6.05	6.66	7.05	5.42	5.15	4.67	4.19	4.54	3.93	4.13	4.01	3.65	3.53	3.77	3.23	3.75	3.66	3.65	3.09	4.01	4.10	
Dominica	13.18	2.93	7.30	4.37	2.91	4.36	14.52	2.90	10.16	13.07	5.79	28.94	25.82	55.42	21.30	8.62	8.76	4.40	8.84	5.92			
Ecuador	1.43	1.13	1.17	1.47	1.25	1.51	1.63	1.70	1.55	2.09	1.57	1.19	1.46	1.35	1.09	1.14	1.19	1.35	1.42	1.64	1.69	1.46	
Estonia	6.05	5.15	8.17	7.41	7.01	6.90	6.56	8.83	8.02	6.01	6.40	7.33	5.46	7.15	7.84	8.06	7.36	6.51	9.27	10.45	11.11	8.15	
Finland	6.09	6.75	6.20	7.10	6.63	6.48	7.13	5.87	5.84	6.13	6.07	5.87	5.79	5.13	4.84	4.79	5.37	4.53	4.58	4.77	4.24	4.24	
France	10.76	10.78	10.66	9.68	9.34	9.03	8.80	8.45	8.28	7.99	6.01	5.79	5.56	5.25	5.63	5.60	5.64	5.43	5.14	5.13	5.33	5.31	
Georgia	0.81			2.56	1.94	2.95	2.07		2.37	1.00	2.69	2.34	2.31	0.76	3.93	4.51	4.77	5.43	7.35	7.64	9.50		4.49
Germany	13.60	12.45	12.95	12.82	13.13	12.57	12.73	12.73	12.54	12.03	11.16	10.67	10.77	9.83	10.00	9.16	8.31	7.82	7.48	7.39	7.49	7.27	
Grenada	4.63	11.07	10.10	9.15	2.73	4.54	4.52	4.51	4.49	3.58	4.46	4.43	4.41	8.76	4.36	6.95	6.93	2.59	1.72	4.30	7.71		
Guatemala					1.03	0.90	0.86	1.06	0.99	1.25	1.10	1.09	1.52	1.36	1.20	1.32	1.26	1.38	1.32	1.12	1.74	1.68	
Guyana	1.05	1.70	2.23	2.23	1.84	2.77	2.25	2.25	0.00	3.34	2.28	2.95	2.67	4.24	3.16	3.67	4.16	3.41	1.86				
Hungary	18.05	16.18	12.55	10.40	6.92	6.29	6.65	6.48	7.61	7.12	6.14	6.76	6.48	6.03	5.47	4.31	4.35	4.83	4.43	4.92	5.59	5.28	4.50
Iceland	5.96	7.65	4.14	3.42	2.70	2.30	6.10	4.41	3.45	2.51	2.51	2.81	2.47	1.83	2.72	3.58	4.66	2.83	3.88	3.27	2.42	2.37	
Ireland							4.42	4.50	4.17	4.65	4.32	4.52	4.04	4.01	3.68	2.78	3.66	3.37	3.30	3.31	4.28	4.32	
Israel	2.41	3.19	2.72	2.57	2.79	2.78	2.99	2.74	2.72	2.39	2.40	2.42	2.54	2.82	2.32	2.11	1.94	1.88	1.91	1.90	2.30	1.91	
Italy			4.84	4.27	4.34	4.43	4.44	3.11	3.28	3.13	3.30	3.42	3.12	3.15	3.35	3.21	3.42	3.43	3.23	3.47	3.59		
Jamaica	4.23	3.56	3.27	4.19	3.57	1.30			3.14	4.07	4.13	3.79	3.23	4.90									
Japan	1.58	1.60	1.55	1.70	1.76	1.73	1.78	1.76	1.73	1.85	1.99	1.89	1.87	1.82	1.88	1.92	1.65	1.64	1.68	1.64	1.83		
Kuwait	1.34	1.41	0.81	1.72	1.03	1.14	0.68	1.13	1.29	0.78	0.93	1.41	1.08	0.90	1.07	0.42	0.41	0.60	0.38			0.46	0.52
Kyrgyzstan	2.43	2.15	2.23	1.92	1.70	1.61	2.01	1.85	1.24	1.78	1.36	1.56	1.64	1.97	1.83	1.95	2.15	2.05	2.42				
Latvia	2.65	3.33	3.19	3.31	2.86	4.24	3.68	3.90	3.87	5.82	4.51	4.03	4.67	4.56	4.70	6.07	7.10	7.83	9.04	10.57	11.46	9.57	11.47
Lithuania	7.83	9.09	8.81	9.03	10.53	13.15	11.95	9.50	11.38	9.49	9.81	10.88	12.78	10.57	11.74	11.82	12.19	12.60	13.17	14.41	14.10	12.11	9.78
Luxembourg	6.56	10.08	17.93	11.78	13.32	13.11	11.24	13.50	14.45	14.58	13.50	10.92	9.20	7.01	6.67	7.03	7.54	6.58	4.84	7.29	5.78	4.44	
Malta	6.95	4.68	4.65	3.41	4.60	4.81	3.84	6.44	2.85	3.55	2.59	5.16	5.37	3.22	4.72	3.08	3.85	5.37	4.76	5.02	6.67		
Mauritius					1.91	2.77	1.89	1.88	2.58	2.65	2.18	2.25	1.70	2.32	2.32	2.86	2.94	2.48	1.94	3.82	4.22	2.35	1.81
Mexico	1.46	1.52	1.56	1.52	1.49	1.60	1.35	1.35	1.36	1.44	1.44	1.40	1.48	1.62	1.76	1.81	1.79	1.85	1.82	1.74	2.15	2.15	
Netherlands	3.86	3.94	3.98	3.99	3.77	3.77	3.57	3.39	3.46	3.21	2.94	3.23	2.71	2.49	2.74	2.39	2.37	2.57	2.33	2.30	2.55	2.64	2.53
New Zealand	1.10	1.06	0.57	0.69	1.04	0.77	1.07	1.13	1.25	1.22	1.51	1.52	1.48	1.57	1.91	1.74	2.22	2.20					
Nicaragua	0.37	0.62	0.52	0.68	0.86	1.03	1.06	1.31	1.27	1.13	1.03	1.27	1.27	1.37	1.30	1.72	1.61	1.69	2.08	3.75	17.31	2.50	
Norway	4.45	5.09	4.99	5.68	3.48	3.80	3.63	3.67	3.38	3.60	3.23	3.41	2.91	3.08	3.24	2.71							
Panama	1.30	1.37	1.50	1.85	2.63	1.42	2.30	2.49	3.03	2.87	2.79	2.69	3.06	2.18	3.13	2.41	2.34	2.42	2.34		1.84	2.57	
Paraguay	1.10	1.18	1.48	1.48	1.39	1.18	0.97	1.30	1.22	1.25	1.14	0.97	0.84	0.69	0.81	1.10	1.04	0.82	0.98	1.06	1.42		
Philippines	0.31	0.31	0.25			0.30	0.32	0.31	0.32	0.32	0.37			0.59		0.44	0.39	0.35	0.39				
Poland	7.38	7.61	7.71	8.51	8.43	8.02	7.57	7.41	7.82	7.08	6.84	6.85	6.44	5.88	6.04	5.18	4.95	5.13	5.28	5.46	6.44	6.27	
Portugal		8.51	9.34				7.57	7.46	7.60	6.88	5.99	5.43	6.21	7.12	7.29	7.21	7.30	6.18	5.59			6.33	
Republic of Korea	0.38	0.36	0.25	0.34	0.40	0.40	0.48	0.58	0.68	0.74	0.86	0.89	0.76	0.87	0.92	1.03	1.12	1.09	1.18	1.25	1.37	1.34	
Republic of Moldova	1.69	1.88	1.64	2.17	2.63	2.32	3.09	2.62	2.46	2.12	2.31	2.53	2.61	3.14	4.23	2.93	3.95	4.03			4.00		
Romania	3.96	3.90	3.80	3.56	3.46	4.07	3.92	3.93	4.20	4.29	4.40	4.54	4.78	5.05	5.60	6.54	6.70	6.58	5.95				
Saint Kitts and Nevis	10.93	10.86	15.16	30.23	2.15	8.59	2.14	6.41	2.13	2.13	2.13	0.00	4.25	2.12	0.00	4.25							

Saint Lucia	6.86	8.04	11.66	10.96	8.46	5.40		8.89	10.60	14.63	6.40	7.53	7.50	4.02	3.43	4.55	8.51	7.34	6.19	6.73			
Saint Vincent and Grena	9.69	8.82	6.19	5.32	6.22	6.25	7.17	6.30	11.76	10.91	16.46	14.71	10.17	14.88	11.22	15.99	21.77	13.34	14.40	28.98	22.36		
Serbia	5.57	5.41	4.94	6.17	7.20	6.95	5.92	6.39	6.56	6.94	6.35	7.03	7.78	7.36	8.19	8.38	8.64	9.24	8.48	7.60	9.85	9.78	7.87
Slovakia	16.30	17.89	18.45	18.77	16.44	16.63	16.59	16.04	15.65	13.91		7.25	9.87	8.49		11.41	11.39	11.90	10.55	9.83	14.24	12.46	10.17
Slovenia	7.64	5.92	4.72	6.27	7.86	9.59	7.15	6.22	7.61	7.39	7.52	8.43	7.73	6.90	7.33	5.77	4.99	5.90	5.18	4.95			
South Africa	2.69	2.93	3.23	3.08	2.89	3.07	3.10	3.26	3.37	3.24	3.12	3.04	2.98	3.08	3.42	3.28	3.43	3.65	3.59	3.78			
Spain	6.74	6.88	6.85	6.05	6.36	5.75	6.00	5.87	5.65	4.80	4.78	4.72	4.74	4.67	4.99	4.63	4.59	4.72	4.56	5.11	5.62	5.58	
Suriname	2.88	4.23	3.75	0.97	2.50	3.24	1.32	1.49	1.47	2.73	1.62	3.02	3.16	2.43									
Sweden	9.29	9.83	8.77	5.80	5.88	5.79	5.78	5.47	5.65	4.67	5.38	5.77	5.17	4.96	4.69	4.72	4.45	4.87	4.49	4.39	4.45	4.29	4.20
Switzerland	6.60	6.76	6.64	5.39	5.52	4.96	4.67	4.99	5.30	4.92	4.92	5.28	5.02	5.07	4.94	5.12	5.13	4.66	4.86	5.35	4.74	5.27	
Thailand		0.20	0.28	0.33	0.40	0.41	0.37	0.42	0.44	0.47	0.54	0.59	0.70	0.87	0.92	0.92	0.99	1.14	1.15		1.36		
Turkey									1.81	1.91	2.17	2.25	2.64	3.16	2.28	2.47	2.90	2.59	2.62	2.84	3.48	3.06	3.08
United Kingdom	13.41	13.18	13.53	12.54	12.03	12.08	11.45	11.42	11.38	11.51	8.43	8.40	8.44	8.11	8.18	7.83	7.87	7.55	6.95	7.93	8.12		
United States of Americ	4.25	4.23	4.22	3.93	3.93	3.24	3.32	3.27	3.24	3.33	3.39	3.40	3.44	3.50	3.61	3.56	3.60	3.61	3.50	3.88	3.87	3.70	
Uruguay	8.00	8.66	9.17	9.62	9.47	7.87	7.80	7.45	7.01	7.71		7.23	5.29	4.35	4.93	5.98	5.40	5.89	5.80	5.59	6.54	6.46	
Uzbekistan				0.75	0.41				0.98	1.00	0.76	0.89	1.34	1.89	3.21	3.15	3.55	3.86	3.47				
Venezuela	0.87	0.86	0.94	1.07	0.99	1.20	1.23	1.21	1.47	1.56	1.34	1.30	1.30	1.58	1.66	2.61							

Data are number of deaths per 100,000 people. LOESS=locally weighted regression. CI=confidence interval.

Supplemental Table 5. Age-standardised PE-related mortality rates in sensitivity analysis

Main Analysis

Year	2001	2002	2003	2004	2005	2006	2007	2008	2009	2010	2011	2012	2013	2014	2015	2016	2017	2018	2019	2020	2021	2022	2023
LOESS smoothed rate	3.49	3.35	3.22	3.10	2.98	2.88	2.78	2.68	2.60	2.55	2.51	2.49	2.48	2.45	2.43	2.41	2.40	2.40	2.39	2.39	2.40	2.41	2.42
Upper 95% CI	3.79	3.57	3.39	3.25	3.13	3.03	2.93	2.84	2.76	2.70	2.67	2.64	2.64	2.60	2.57	2.56	2.54	2.53	2.53	2.55	2.61	2.69	2.80
Lower 95% CI	3.20	3.14	3.06	2.95	2.84	2.72	2.62	2.53	2.44	2.40	2.35	2.34	2.32	2.30	2.29	2.27	2.26	2.26	2.25	2.23	2.19	2.12	2.04

Sensitivity Analysis

Used ICD-10 codes: I26x, O88.2 (code O88 if sub-codes not recorded)

Year	2001	2002	2003	2004	2005	2006	2007	2008	2009	2010	2011	2012	2013	2014	2015	2016	2017	2018	2019	2020	2021	2022	2023
LOESS smoothed rate	2.52	2.43	2.34	2.26	2.18	2.11	2.03	1.96	1.91	1.87	1.84	1.84	1.83	1.82	1.81	1.80	1.80	1.80	1.81	1.81	1.83	1.84	1.86
Upper 95% CI	2.74	2.59	2.47	2.37	2.29	2.22	2.15	2.08	2.03	1.99	1.96	1.95	1.95	1.93	1.92	1.91	1.91	1.90	1.91	1.93	1.98	2.05	2.15
Lower 95% CI	2.30	2.27	2.22	2.15	2.07	1.99	1.92	1.85	1.79	1.75	1.72	1.72	1.71	1.71	1.70	1.69	1.70	1.70	1.70	1.69	1.67	1.63	1.57

Data are number of deaths per 100,000 people. LOESS=locally weighted regression. CI=confidence interval.

Diffrence

Year	2001	2002	2003	2004	2005	2006	2007	2008	2009	2010	2011	2012	2013	2014	2015	2016	2017	2018	2019	2020	2021	2022	2023
LOESS smoothed rate	-0.97	-0.93	-0.88	-0.84	-0.81	-0.77	-0.74	-0.72	-0.69	-0.68	-0.67	-0.66	-0.65	-0.63	-0.62	-0.61	-0.60	-0.59	-0.59	-0.58	-0.57	-0.57	-0.56
Upper 95% CI	-1.05	-0.98	-0.92	-0.88	-0.84	-0.81	-0.78	-0.75	-0.73	-0.72	-0.71	-0.69	-0.69	-0.67	-0.66	-0.65	-0.64	-0.63	-0.62	-0.62	-0.62	-0.64	-0.65
Lower 95% CI	-0.90	-0.87	-0.84	-0.81	-0.77	-0.73	-0.70	-0.68	-0.65	-0.64	-0.63	-0.62	-0.61	-0.60	-0.58	-0.57	-0.57	-0.56	-0.55	-0.54	-0.52	-0.50	-0.47

Supplementary Table 6. Age-standardised pulmonary embolism-related mortality rates for 73 countries for male

Year	2001	2002	2003	2004	2005	2006	2007	2008	2009	2010	2011	2012	2013	2014	2015	2016	2017	2018	2019	2020	2021	2022	2023
LOESS smoothed rate	3.56	3.42	3.28	3.15	3.03	2.92	2.81	2.70	2.62	2.56	2.52	2.51	2.50	2.48	2.45	2.44	2.44	2.44	2.45	2.47	2.49	2.52	2.56
Upper 95% CI	3.87	3.64	3.46	3.31	3.18	3.08	2.97	2.87	2.79	2.73	2.69	2.67	2.67	2.64	2.61	2.60	2.59	2.59	2.60	2.64	2.72	2.82	2.96
Lower 95% CI	3.25	3.19	3.10	2.99	2.87	2.75	2.64	2.54	2.45	2.40	2.36	2.35	2.33	2.32	2.30	2.29	2.29	2.30	2.31	2.30	2.27	2.22	2.15
Antigua and Barbuda	14.83	12.05	21.43	3.46	7.70	8.90	18.23	19.75	17.06	11.22	23.54	35.15	6.40	23.40	11.14	14.91	25.18	7.37	9.64	10.60	17.44		
Argentina	3.61	3.57	3.34	3.27	3.16	3.03	3.10	2.81	2.83	2.81	2.99	2.78	2.98	3.03	2.96	3.16	3.18	2.51	2.63	2.51	2.95	2.89	
Armenia						5.43	3.63	4.70	6.04	6.66	7.11	5.66	1.72	2.99	2.86	3.47	3.02	5.97	5.36	8.53	7.88	5.17	
Australia	1.74	2.07	1.96	1.80		1.46	1.44	1.60	1.50	1.37	1.39	1.19	1.31	1.52	1.45	1.36	1.25	1.31	1.35	1.41	1.38	1.36	1.23
Austria		4.13	3.80	3.48	3.83	3.17	3.47	2.60	2.53	2.99	2.43	2.24	2.23	2.14	2.55	2.66	2.59	2.54	2.40	2.08	2.24	1.95	1.96
Bahamas	2.61	9.27	0.73	2.62	7.53	3.17	5.82	6.87	5.69	11.15	8.14	9.48	8.96	6.92	9.95								
Belgium	5.51	4.90	5.13	4.97	4.78	3.80	3.93	3.43	3.91	3.61	3.45	3.11	2.99	3.11	2.87	3.21	2.64	2.75	2.39	2.53	2.66		
Belize	3.08	5.63	6.22	1.41	3.77	8.84	2.11	2.81	0.73	5.59	7.03	3.67	3.35	4.45	1.29	4.59							
Brazil	5.25	4.89	4.83	4.68	4.38	4.43	4.24	4.17	4.09	3.99	4.24	4.20	4.28	4.26	4.52	4.40	3.96	3.80	3.92	3.12	3.78		
Canada	1.92	1.83	1.99	1.84	1.80	1.35	1.36	1.38	1.63	1.49	1.42	1.31	1.30	1.28	1.33	1.35	1.27	1.37	1.30	1.48	1.41	1.35	
Chile	1.77	2.02	1.90	1.94	1.93	1.44	1.42	1.38	1.83	1.85	1.28	1.17	1.63	1.78	1.60	1.88	1.85	2.13	2.31	2.14	2.57		
Colombia	4.06	4.34	4.63	4.22	5.08	4.51	3.86	3.65	3.39	3.37	3.78	3.76	3.00	2.48	2.66	2.27	2.07	2.06	2.00	2.17	3.41		
Costa Rica	5.96	3.44	3.44	2.98	2.08	1.93	2.91	2.09	1.84	3.00	2.61	2.54	2.44	1.85	1.61	2.23	1.65	2.53	2.93	2.44	2.45	2.00	
Croatia	9.48	9.57	9.68	9.67	7.58	7.44	8.07	6.09	3.39	1.96	2.72	2.42	1.97	1.64	2.42	1.99	2.28	1.62	1.14	1.16	1.35		
Cuba	3.52	2.83	2.67	2.99	3.06	2.72	2.51	2.45	2.42	2.51	1.92	2.32	2.43	2.64	2.20	2.51	3.59	3.88	4.50	5.00	12.77		
Cyprus				0.87	1.02	0.96	1.07	0.92	0.58	0.98	0.94	0.89	0.92	0.73	1.50	0.91	1.29	0.68	0.94	0.82	1.40	0.98	
Czech Republic	12.84	13.22	13.78	11.49	11.31	11.78	11.06	11.94	12.78	11.61	9.05	8.17	6.85	6.39	7.41	6.01	6.13	6.16	5.86	6.12	6.88	5.64	4.94
Denmark	3.49	3.73	4.18	3.78	3.12	2.47	3.14	2.29	2.43	2.23	1.74	2.22	1.84	1.97	1.91	1.85	1.92	1.85	1.66	1.63	1.94	1.87	
Dominica	11.17	2.74	5.46	2.52	0.00	3.34	14.16	2.27	10.61	7.91	5.64	22.32	15.63	56.48	18.03	7.22	2.35	4.09	2.04	2.14			
Ecuador	2.79	2.35	2.07	2.69	1.85	3.06	2.78	2.83	2.56	3.28	2.36	1.70	2.06	1.74	1.37	1.49	1.51	1.91	1.86	2.10	2.19	1.62	
Estonia	4.28	4.16	7.36	5.44	5.52	4.70	4.25	5.83	5.34	3.99	3.12	2.96	2.77	4.15	3.10	4.44	4.08	3.41	5.51	6.75	5.67	3.15	
Finland	3.28	3.44	3.31	4.10	3.46	3.55	3.66	3.12	2.63	2.94	2.91	2.64	2.71	2.31	2.32	2.02	2.96	2.25	1.80	2.00	1.76	1.85	
France	5.16	5.22	5.04	4.65	4.45	4.27	4.02	3.74	3.73	3.48	2.56	2.50	2.33	2.24	2.22	2.25	2.19	2.15	1.89	2.08	2.15	2.20	
Georgia	0.81			2.37	2.62	3.09	2.05		2.27	0.80	2.05	2.05	1.64	0.61	2.65	3.37	4.02	3.43	5.52	6.91	7.93		4.08
Germany	7.27	6.76	6.97	6.73	6.69	6.07	6.19	6.02	5.67	5.22	4.89	4.54	4.51	4.26	4.20	3.89	3.55	3.40	3.24	3.30	3.30	3.03	
Grenada	0.00	5.45	14.63	8.38	4.71	6.05	1.92	1.95	3.35	6.78	3.29	0.00	4.77	8.43	2.80	4.53	3.84	3.07	3.33	4.05	2.57		
Guatemala					1.85	1.83	1.75	2.19	1.83	1.99	1.96	2.10	2.47	2.41	2.02	2.35	2.00	2.36	2.06	1.99	3.15	3.07	
Guyana	1.83	4.16	3.42	4.43	4.38	6.26	1.96	1.89	0.00	4.10	4.25	3.56	4.12	5.89	4.88	5.90	6.40	4.01	2.36				
Hungary	11.85	10.65	7.87	6.58	4.73	4.02	4.07	3.90	4.37	4.14	3.52	3.87	3.31	3.23	2.98	2.04	2.32	3.05	2.22	3.19	3.73	3.12	2.49
Iceland	6.23	4.88	2.06	1.05	2.22	1.05	2.23	1.29	2.67	2.15	1.17	1.31	0.80	0.92	0.84	1.67	3.39	1.66	2.34	1.75	1.63	1.98	
Ireland							3.02	3.17	2.63	3.50	2.94	3.14	2.30	2.57	2.07	1.78	2.35	1.93	2.13	1.98	2.53	2.93	
Israel	1.62	2.08	2.01	1.73	1.93	1.59	1.59	1.65	1.61	1.19	1.19	1.32	1.33	1.79	1.26	1.04	1.05	1.30	1.14	0.98	1.37	1.23	
Italy			1.96	1.69	1.62	1.65	1.57	1.09	1.10	1.08	1.06	1.05	0.92	0.99	0.99	0.95	0.92	0.89	0.91	1.01	0.99		
Jamaica	5.14	4.71	4.26	5.35	4.95	1.40			3.18	4.20	5.53	5.34	4.30	5.27									
Japan	0.82	0.77	0.70	0.79	0.80	0.76	0.77	0.73	0.69	0.66	0.76	0.66	0.66	0.65	0.71	0.64	0.51	0.54	0.53	0.56	0.55		
Kuwait	2.96	3.51	3.18	4.94	1.64	3.04	0.92	2.54	1.39	1.95	1.76	3.41	2.03	1.27	2.82	0.68	0.58	0.95	0.92			1.09	0.43
Kyrgyzstan	4.27	3.77	4.94	2.93	2.80	2.56	3.67	3.31	2.43	2.92	1.97	2.17	2.81	3.09	2.96	3.20	3.49	2.90	4.02				

Latvia	1.81	3.76	2.66	3.27	1.80	3.39	2.99	2.82	2.95	4.74	3.59	3.21	3.52	3.49	3.49	4.07	4.46	3.78	5.69	6.43	6.40	4.62	7.04
Lithuania	6.78	7.65	6.84	6.89	8.95	10.48	9.71	7.48	9.02	6.63	6.58	7.99	9.18	6.47	7.57	5.94	6.44	6.76	7.33	7.53	7.85	6.86	6.32
Luxembourg	4.15	5.34	7.68	7.49	8.35	7.18	7.06	7.44	6.07	8.16	7.56	6.33	5.01	3.42	2.33	2.75	3.14	3.40	2.34	4.94	1.70	2.57	
Malta	4.22	4.01	2.45	2.42	3.51	1.64	2.49	3.44	1.34	1.60	0.00	2.47	3.44	1.68	2.49	0.66	1.06	3.27	2.19	2.90	2.53		
Mauritius					1.87	3.47	1.89	1.47	2.80	2.22	2.86	3.25	1.65	2.29	2.83	2.29	3.00	1.86	1.87	3.02	3.53	1.36	1.05
Mexico	1.99	2.08	2.12	2.02	2.03	2.01	1.69	1.66	1.64	1.67	1.81	1.64	1.68	1.96	2.10	2.07	2.03	2.11	2.00	2.00	2.53	2.38	
Netherlands	2.10	2.12	2.11	2.13	2.08	2.11	2.15	1.58	1.84	1.57	1.31	1.46	1.51	1.27	1.38	1.14	1.19	1.19	1.09	1.14	1.16	1.24	1.14
New Zealand	0.75	0.68	0.47	0.34	0.60	0.46	0.60	0.49	0.54	0.64	0.98	1.08	0.70	0.82	0.90	1.15	1.22	1.17					
Nicaragua	0.18	1.11	1.09	1.43	2.05	2.29	2.30	1.90	2.15	2.05	1.98	2.21	2.00	1.77	2.24	3.17	2.65	2.63	3.39	7.33	29.40	3.98	
Norway	2.16	2.38	2.31	2.85	1.50	1.98	1.71	2.15	1.47	1.51	1.53	1.96	1.25	1.58	1.89	1.30							
Panama	1.57	3.40	2.78	3.37	5.10	2.36	3.70	3.59	4.24	4.27	4.16	3.90	3.97	2.66	3.56	2.33	2.77	2.69	2.69		1.71	2.45	
Paraguay	2.89	2.28	3.20	3.69	2.49	2.21	2.21	2.21	2.15	2.10	2.09	1.17	0.94	1.43	0.77	1.29	1.41	1.09	1.11	1.17	2.25		
Philippines	0.94	0.80	0.80			0.79	0.74	0.59	0.79	0.68	0.95			1.43		0.93	0.62	0.59	0.63				
Poland	6.87	6.70	6.76	7.46	7.32	6.52	6.27	5.66	6.14	5.53	5.02	5.03	4.62	4.01	4.01	3.49	3.20	3.43	3.29	3.44	3.99	3.76	
Portugal		5.09	5.58				3.79	3.70	3.47	3.24	2.76	2.00	2.62	3.17	3.27	2.84	2.94	2.20	2.09			2.41	
Republic of Korea	0.45	0.45	0.22	0.38	0.32	0.33	0.45	0.39	0.56	0.58	0.50	0.60	0.48	0.55	0.56	0.56	0.57	0.49	0.61	0.62	0.66	0.57	
Republic of Moldova	1.89	2.10	2.18	2.86	3.08	2.61	4.04	2.72	3.16	2.33	2.38	2.51	2.31	2.73	4.11	3.10	3.96	3.60			3.15		
Romania	3.16	2.95	2.96	2.81	2.62	3.11	2.88	2.75	2.88	2.99	2.88	2.98	2.98	3.23	3.58	4.15	4.06	3.97	3.52				
Saint Kitts and Nevis	26.08	9.03	19.35	33.21	0.00	3.73	0.00	10.20	5.94	0.00	4.24	0.00	5.38	0.00	0.00	6.53							
Saint Lucia	10.93	8.91	12.72	19.43	14.63	3.70		12.29	15.37	21.42	12.30	6.07	9.78	3.57	8.07	2.65	6.29	9.80	2.67	5.47			
Saint Vincent and Grena	13.95	15.50	6.63	9.30	4.01	7.54	7.98	8.18	10.85	10.27	21.21	13.97	10.23	15.31	12.50	14.66	14.64	11.65	10.12	28.60	18.23		
Serbia	3.77	3.61	2.92	3.76	4.66	4.35	3.20	3.58	3.28	3.50	3.30	3.39	3.73	3.34	4.00	4.05	4.15	4.07	3.94	3.62	4.21	4.09	3.49
Slovakia	13.59	15.27	15.99	15.73	14.43	13.01	12.90	11.38	11.73	10.12		5.00	7.01	5.72		7.63	7.31	8.03	6.83	6.42	9.78	8.15	6.13
Slovenia	5.17	4.58	2.39	4.27	4.66	6.16	4.06	3.45	3.25	3.79	3.13	3.54	3.17	2.86	2.84	2.24	1.80	2.12	1.96	1.44			
South Africa	3.74	4.14	4.71	4.01	3.86	4.02	4.03	3.91	4.46	3.81	3.92	3.70	3.60	3.71	4.14	4.03	3.96	4.18	3.80	3.99			
Spain	3.74	3.64	3.56	2.99	3.17	2.84	2.85	2.63	2.69	2.18	2.05	1.98	1.91	1.88	2.04	1.94	1.95	2.03	1.91	2.10	2.29	2.43	
Suriname	3.34	8.51	5.31	1.20	2.73	3.96	2.94	1.13	1.47	4.27	1.59	2.93	1.98	2.81									
Sweden	4.41	4.18	4.31	2.85	2.81	2.59	2.53	2.55	2.39	2.10	2.72	2.75	2.44	2.19	2.31	2.30	2.12	2.42	1.99	2.20	2.38	1.76	1.98
Switzerland	2.75	3.07	2.77	2.31	2.67	2.09	1.89	2.18	2.16	1.80	2.12	1.92	1.95	1.96	1.84	1.98	1.86	1.80	2.07	2.13	1.88	1.98	
Thailand		0.19	0.25	0.27	0.40	0.36	0.29	0.34	0.34	0.39	0.40	0.42	0.49	0.56	0.57	0.59	0.61	0.69	0.64		0.84		
Turkey									2.20	2.21	2.41	2.38	2.82	3.21	2.30	2.46	2.80	2.37	2.31	2.58	2.89	2.42	2.38
United Kingdom	6.67	6.31	6.29	5.95	5.87	5.77	5.61	5.39	5.41	5.47	4.00	3.87	3.89	4.05	4.02	3.93	3.89	3.87	3.43	4.35	4.27		
United States of Americ	2.85	2.77	2.85	2.62	2.62	2.16	2.20	2.18	2.16	2.25	2.31	2.29	2.32	2.35	2.42	2.40	2.39	2.40	2.29	2.62	2.61	2.35	
Uruguay	4.53	5.44	5.23	4.82	5.26	4.28	4.58	3.40	4.17	4.16		4.18	2.13	2.06	1.96	2.60	2.49	2.80	2.40	2.57	3.48	2.34	
Uzbekistan				1.27	0.67				1.76	1.73	1.30	1.60	2.23	3.25	5.95	5.47	6.04	6.73	5.96				
Venezuela	1.59	1.38	1.55	1.75	1.53	1.89	1.87	1.59	1.99	1.85	1.77	1.66	1.50	1.94	1.95	3.25							

Data are number of deaths per 100,000 people. LOESS=locally weighted regression. CI=confidence interval.

Supplementary Table 7. Age-standardised pulmonary embolism-related mortality rates for 73 countries for female

Year	2001	2002	2003	2004	2005	2006	2007	2008	2009	2010	2011	2012	2013	2014	2015	2016	2017	2018	2019	2020	2021	2022	2023
LOESS smoothed rate	3.43	3.29	3.17	3.05	2.93	2.83	2.73	2.65	2.57	2.52	2.48	2.46	2.45	2.42	2.40	2.38	2.36	2.34	2.32	2.31	2.30	2.29	2.28
Upper 95% CI	3.71	3.50	3.33	3.19	3.08	2.98	2.88	2.79	2.72	2.67	2.64	2.61	2.60	2.56	2.54	2.52	2.49	2.47	2.46	2.46	2.50	2.56	2.65
Lower 95% CI	3.15	3.09	3.01	2.90	2.79	2.68	2.59	2.50	2.42	2.37	2.33	2.31	2.29	2.27	2.26	2.23	2.22	2.21	2.19	2.15	2.09	2.01	1.91
Antigua and Barbuda	9.03	11.34	12.07	13.25	20.00	11.06	8.35	15.83	1.82	5.86	7.74	15.04	9.39	17.84	7.49	8.40	9.89	7.13	11.48	11.89	10.36		
Argentina	3.23	2.98	3.02	2.89	2.91	2.79	2.90	2.61	2.56	2.81	2.88	2.80	2.82	2.81	2.96	3.09	3.03	2.82	2.50	2.56	2.90	2.90	
Armenia						4.51	3.47	2.85	3.85	5.07	4.18	3.94	2.72	3.39	2.56	3.25	4.24	4.23	6.18	6.72	6.29	4.63	
Australia	1.90	2.02	2.16	2.07		1.74	1.77	1.67	1.43	1.38	1.39	1.44	1.34	1.48	1.43	1.33	1.30	1.14	1.50	1.27	1.34	1.24	1.06
Austria		4.38	4.09	3.44	4.03	3.73	3.45	2.81	3.03	2.67	2.46	2.35	2.27	2.14	2.54	2.36	2.35	2.72	2.00	1.96	1.65	1.54	1.70
Bahamas	4.09	11.67	4.25	5.99	5.77	2.68	6.85	6.26	6.81	8.50	8.73	9.42	6.44	13.30	10.87								
Belgium	5.29	5.70	5.60	4.85	4.43	4.44	4.52	3.65	3.39	3.70	2.78	3.34	3.28	2.95	3.20	2.61	2.58	2.56	2.33	2.11	2.34		
Belize	9.21	6.96	3.91	7.52	1.46	4.11	7.94	6.41	3.62	3.67	1.89	5.23	7.65	3.19	5.64	2.66							
Brazil	5.20	5.31	5.12	4.94	4.57	4.76	4.69	4.41	4.51	4.48	4.63	4.68	4.61	4.75	4.84	4.94	4.59	4.37	4.40	3.37	3.97		
Canada	2.10	2.09	2.09	1.85	1.90	1.33	1.41	1.51	1.43	1.31	1.45	1.42	1.25	1.29	1.37	1.40	1.26	1.28	1.27	1.46	1.43	1.44	
Chile	1.60	1.69	1.53	1.80	1.53	1.63	1.82	1.57	1.79	1.80	1.45	1.30	1.64	1.92	1.91	1.97	2.13	1.91	2.31	2.06	2.26		
Colombia	4.15	4.63	4.77	4.86	5.37	4.80	5.01	4.38	4.33	3.95	4.04	3.86	3.16	2.90	2.87	2.71	2.55	2.62	2.39	2.49	3.48		
Costa Rica	4.90	4.27	2.83	3.34	2.26	2.75	2.96	2.44	2.36	2.85	2.50	2.72	2.31	2.55	1.86	2.56	2.38	2.50	3.49	1.91	2.19	1.75	
Croatia	7.56	7.56	7.58	7.17	6.08	5.61	5.68	4.56	2.66	2.10	2.26	1.83	1.58	1.83	1.66	1.62	2.08	1.55	1.14	0.96	0.98		
Cuba	3.19	2.80	2.69	2.92	2.63	2.83	2.13	2.14	1.78	2.35	2.06	2.26	2.33	2.49	2.08	2.09	2.95	3.25	3.78	3.85	8.56		
Cyprus				1.21	1.22	1.38	0.38	1.16	1.45	0.94	0.70	0.63	0.57	0.91	1.51	0.95	0.73	0.74	1.52	0.84	1.04	1.82	
Czech Republic	11.25	10.45	11.85	10.48	10.26	10.59	9.23	10.39	10.99	10.30	7.55	7.05	6.56	5.29	5.83	5.28	5.81	4.71	4.90	5.16	4.76	4.24	4.00
Denmark	2.88	2.95	3.04	3.70	2.63	2.68	2.02	1.97	2.28	1.85	2.29	1.64	1.72	1.53	1.75	1.30	1.58	1.67	1.52	1.17	1.68	1.63	
Dominica	8.09	1.22	8.20	3.78	3.75	4.28	11.54	1.76	5.76	12.84	6.20	25.20	28.81	42.33	16.62	5.12	10.06	1.06	10.54	5.94			
Ecuador	2.16	1.49	1.68	1.98	1.75	1.69	2.06	2.13	1.80	2.48	1.94	1.47	1.83	1.62	1.32	1.30	1.30	1.30	1.37	1.54	1.49	1.45	
Estonia	3.47	2.55	3.40	4.10	3.25	3.37	2.81	4.73	3.86	2.57	2.94	3.71	2.62	2.91	3.56	2.82	2.30	2.62	2.80	3.50	4.13	3.39	
Finland	3.23	3.55	3.14	3.31	3.29	3.00	3.02	2.69	2.80	3.05	2.58	2.83	2.49	2.24	2.08	2.17	2.27	1.78	2.22	2.19	1.90	1.74	
France	4.78	4.80	4.71	4.19	3.97	3.75	3.60	3.35	3.19	3.06	2.39	2.15	2.03	1.85	2.02	1.87	1.93	1.88	1.78	1.73	1.74	1.74	
Georgia	0.59			1.75	0.92	1.33	0.87		1.30	0.53	1.96	1.26	1.37	0.40	2.15	2.44	2.23	2.80	3.40	3.11	4.14		1.91
Germany	6.38	5.61	5.74	5.66	5.59	5.38	5.05	4.99	4.93	4.63	4.20	3.95	4.08	3.51	3.61	3.27	2.92	2.81	2.61	2.57	2.44	2.39	
Grenada	9.61	15.09	7.99	9.32	2.34	3.57	6.38	5.89	3.17	0.00	4.22	8.14	2.52	7.76	3.79	7.14	7.54	2.15	0.00	1.93	10.18		
Guatemala					2.49	1.79	1.80	2.05	1.89	2.60	2.19	1.89	2.87	2.51	2.15	2.22	2.30	2.26	2.32	1.73	2.74	2.89	
Guyana	0.85	2.64	4.65	2.99	2.19	3.12	4.11	4.69	0.00	5.16	2.57	4.91	3.39	5.75	3.94	4.55	4.48	5.14	2.05				
Hungary	9.58	8.42	6.58	5.41	3.47	3.24	3.67	3.15	3.73	3.40	2.82	3.15	3.03	2.58	2.42	2.01	2.00	2.05	1.96	1.91	2.17	2.08	1.79
Iceland	3.29	4.30	2.50	3.21	1.27	2.20	4.99	3.94	1.69	0.98	1.62	1.40	1.72	0.92	1.94	2.74	2.38	2.13	2.00	1.98	1.29	1.00	
Ireland							3.53	3.34	3.03	3.08	2.94	2.98	3.03	2.51	2.79	1.83	2.11	2.36	2.00	2.01	2.56	2.43	
Israel	2.08	2.65	2.21	2.09	2.13	2.16	2.37	2.06	2.00	1.87	1.68	1.69	1.80	1.74	1.60	1.51	1.43	1.18	1.22	1.31	1.44	1.13	
Italy			1.94	1.70	1.74	1.66	1.64	1.11	1.16	1.05	1.16	1.17	1.04	1.01	1.08	0.99	1.12	1.11	1.01	1.01	1.04		
Jamaica	5.94	4.76	4.38	5.45	4.57	1.93			4.63	5.66	4.89	4.62	3.79	6.68									
Japan	0.82	0.83	0.79	0.80	0.80	0.75	0.78	0.75	0.72	0.74	0.76	0.74	0.70	0.66	0.61	0.65	0.56	0.53	0.51	0.51	0.55		
Kuwait	7.95	7.82	4.29	7.12	4.47	5.00	2.64	5.25	5.28	3.25	3.76	4.62	3.61	2.60	4.06	0.91	2.02	2.09	1.15			1.27	2.03
Kyrgyzstan	3.04	2.61	2.26	2.41	2.03	2.21	2.20	2.27	1.31	1.97	1.79	1.86	1.56	2.05	1.69	1.86	2.20	2.30	2.14				

Latvia	1.55	1.35	1.46	1.38	1.67	1.89	1.74	2.14	1.93	2.64	1.74	1.74	1.96	1.50	1.61	1.94	2.66	3.38	2.94	3.56	3.86	3.75	3.26
Lithuania	4.18	4.76	4.60	4.62	4.53	5.88	5.50	4.21	4.84	4.15	4.21	3.93	4.64	4.44	4.06	4.98	4.16	4.76	4.61	5.18	4.54	4.00	3.21
Luxembourg	3.61	5.84	10.81	5.73	6.74	7.00	5.39	7.92	8.10	7.31	6.36	6.12	5.04	3.39	3.98	3.94	4.85	3.48	2.75	3.29	3.75	1.80	
Malta	5.63	2.38	3.67	1.79	2.23	3.66	1.93	4.39	1.24	2.23	2.40	2.62	1.79	1.54	2.83	1.88	2.59	1.71	1.95	2.01	4.12		
Mauritius					2.25	2.67	2.42	2.23	2.56	3.20	1.56	1.51	1.40	1.88	1.45	2.48	1.98	2.04	1.33	3.22	3.01	2.14	1.81
Mexico	2.10	2.07	2.09	2.00	1.83	2.03	1.66	1.68	1.68	1.82	1.69	1.72	1.79	1.81	1.91	2.01	1.92	1.91	1.89	1.68	1.99	2.07	
Netherlands	2.47	2.47	2.63	2.49	2.24	2.11	1.96	2.06	1.91	1.79	1.76	1.90	1.42	1.33	1.48	1.31	1.12	1.24	1.10	1.05	1.23	1.20	1.15
New Zealand	0.70	0.83	0.31	0.52	0.77	0.55	0.89	0.90	0.82	0.90	0.87	0.74	1.02	0.95	1.44	0.92	1.36	1.37					
Nicaragua	0.97	1.58	1.21	1.44	1.50	2.01	2.21	3.06	2.54	2.02	1.69	2.26	2.24	2.42	2.15	2.60	2.47	2.59	3.31	4.64	21.94	3.62	
Norway	1.99	2.31	2.35	2.32	1.72	1.56	1.82	1.66	1.63	1.77	1.51	1.25	1.29	1.39	1.39	1.39							
Panama	2.71	1.65	2.39	2.40	3.65	2.03	2.84	3.22	3.75	3.44	3.00	2.79	3.20	2.39	3.34	2.63	2.20	2.24	1.99		1.68	2.25	
Paraguay	2.02	2.72	2.82	2.59	2.84	2.15	1.43	2.12	1.90	1.92	1.51	1.71	1.51	0.68	1.42	1.66	1.38	1.08	1.43	1.45	1.64		
Philippines	0.59	0.72	0.48			0.63	0.53	0.64	0.65	0.70	0.61			0.92		0.73	0.65	0.60	0.66				
Poland	4.24	4.47	4.35	4.55	4.38	4.22	3.71	3.78	3.80	3.26	3.23	3.10	2.87	2.61	2.73	2.19	2.06	2.06	2.23	2.25	2.48	2.42	
Portugal		4.34	4.29				3.18	3.17	3.38	2.79	2.35	2.34	2.38	2.96	2.76	2.85	2.86	2.62	2.16			2.47	
Republic of Korea	0.43	0.38	0.26	0.33	0.45	0.39	0.42	0.56	0.57	0.58	0.73	0.66	0.53	0.57	0.61	0.66	0.69	0.64	0.58	0.59	0.65	0.62	
Republic of Moldova	1.17	1.32	0.92	1.18	1.71	1.42	1.47	1.66	1.09	1.20	1.30	1.52	1.67	1.90	2.34	1.30	1.94	2.15			2.17		
Romania	2.54	2.51	2.27	2.11	2.04	2.34	2.20	2.23	2.31	2.21	2.16	2.31	2.29	2.31	2.42	2.78	2.92	2.82	2.55				
Saint Kitts and Nevis	4.83	7.24	14.63	28.19	3.73	14.36	2.14	5.23	0.00	5.98	0.00	0.00	3.26	3.26	0.00	0.00							
Saint Lucia	8.94	9.55	18.64	12.63	9.26	10.19		11.90	9.60	11.86	3.47	8.42	6.20	4.15	1.08	6.10	8.59	5.78	9.40	7.38			
Saint Vincent and Grena	9.11	5.08	7.39	3.97	7.20	5.04	6.65	5.13	12.99	10.24	9.92	15.64	9.50	12.10	7.57	14.02	24.70	11.80	14.68	17.45	18.57		
Serbia	3.01	2.94	2.80	3.32	3.63	3.56	3.12	3.28	3.47	3.51	2.97	3.66	3.65	3.58	3.58	3.69	3.81	4.06	3.43	3.02	3.98	4.12	3.17
Slovakia	11.71	12.05	11.65	11.90	9.80	10.45	10.11	9.98	9.03	7.98		4.07	5.31	4.60		5.66	5.76	5.41	5.16	4.38	6.09	5.20	4.50
Slovenia	4.70	3.28	2.93	3.27	4.35	4.65	3.70	3.09	3.90	3.35	3.55	3.63	3.32	2.89	3.00	2.26	2.12	2.23	2.04	1.95			
South Africa	4.28	4.45	4.87	4.82	4.29	4.59	4.53	4.84	4.77	4.78	4.40	4.36	4.20	4.21	4.60	4.36	4.58	4.62	4.67	4.81			
Spain	2.95	2.98	2.97	2.66	2.79	2.40	2.56	2.57	2.19	1.89	1.92	1.90	1.94	1.89	1.90	1.72	1.70	1.68	1.65	1.86	2.08	1.93	
Suriname	4.03	4.62	4.77	1.57	3.87	4.22	1.78	2.52	2.41	2.50	2.09	4.34	5.65	3.16									
Sweden	4.00	4.42	3.52	2.28	2.35	2.22	2.11	2.13	2.36	1.78	2.04	2.19	1.98	1.85	1.74	1.68	1.73	1.78	1.78	1.46	1.55	1.76	1.64
Switzerland	3.39	3.11	3.10	2.59	2.40	2.20	2.06	2.19	2.48	2.25	1.92	2.33	1.95	2.12	2.10	2.14	2.06	1.87	1.66	2.05	1.84	1.81	
Thailand		0.25	0.34	0.42	0.43	0.44	0.43	0.45	0.50	0.46	0.56	0.61	0.69	0.87	0.87	0.82	0.87	0.97	1.00		1.05		
Turkey									1.95	2.07	2.39	2.48	2.87	3.41	2.46	2.62	3.06	2.79	2.70	2.66	3.26	2.82	2.77
United Kingdom	6.73	6.80	6.99	6.33	5.97	6.10	5.61	5.62	5.64	5.56	4.23	4.16	4.19	3.92	3.86	3.80	3.80	3.68	3.40	3.93	4.01		
United States of Americ	3.05	3.05	2.93	2.70	2.67	2.23	2.26	2.21	2.19	2.19	2.20	2.18	2.18	2.21	2.26	2.23	2.26	2.21	2.16	2.42	2.32	2.20	
Uruguay	5.00	4.90	5.19	5.77	5.43	4.65	3.98	4.22	3.43	4.05		3.51	3.23	2.04	2.86	2.93	2.97	3.12	3.08	2.56	2.97	3.40	
Uzbekistan				1.08	0.45				1.37	1.38	1.04	1.13	1.71	2.31	3.68	3.75	3.97	3.94	3.48				
Venezuela	1.63	1.60	1.59	1.72	1.68	1.91	1.97	1.99	2.15	2.43	1.91	1.80	1.82	2.12	2.18	3.23							

Data are number of deaths per 100,000 people. LOESS=locally weighted regression. CI=confidence interval.

Supplementary Table 8. Age-standardised pulmonary embolism-related mortality rates for 73 countries by geography

(A) Northern America

Year	2001	2002	2003	2004	2005	2006	2007	2008	2009	2010	2011	2012	2013	2014	2015	2016	2017	2018	2019	2020	2021	2022	2023
LOESS smoothed rate	2.99	2.82	2.66	2.53	2.41	2.31	2.23	2.16	2.11	2.11	2.13	2.14	2.17	2.19	2.20	2.21	2.23	2.24	2.25	2.26	2.27	2.28	NA
Upper 95% CI	3.31	3.05	2.85	2.69	2.57	2.48	2.40	2.34	2.30	2.29	2.32	2.32	2.34	2.37	2.37	2.38	2.39	2.39	2.40	2.43	2.49	2.58	NA
Lower 95% CI	2.66	2.58	2.48	2.36	2.25	2.14	2.05	1.98	1.92	1.93	1.95	1.97	2.00	2.00	2.02	2.04	2.06	2.08	2.10	2.09	2.05	1.99	NA

(B) Latin America and the Caribbean

Year	2001	2002	2003	2004	2005	2006	2007	2008	2009	2010	2011	2012	2013	2014	2015	2016	2017	2018	2019	2020	2021	2022	2023
LOESS smoothed rate	3.75	3.65	3.56	3.47	3.39	3.31	3.23	3.17	3.14	3.11	3.12	3.13	3.15	3.15	3.14	3.12	3.10	3.08	3.05	3.01	2.97	2.93	NA
Upper 95% CI	4.20	3.97	3.80	3.69	3.62	3.55	3.48	3.43	3.38	3.35	3.35	3.37	3.39	3.37	3.36	3.34	3.32	3.29	3.26	3.27	3.32	3.39	NA
Lower 95% CI	3.30	3.33	3.31	3.24	3.15	3.07	2.98	2.92	2.90	2.87	2.89	2.89	2.92	2.92	2.91	2.90	2.89	2.87	2.84	2.76	2.63	2.46	NA

(C) Western Europe

Year	2001	2002	2003	2004	2005	2006	2007	2008	2009	2010	2011	2012	2013	2014	2015	2016	2017	2018	2019	2020	2021	2022	2023
LOESS smoothed rate	5.24	4.97	4.70	4.45	4.21	3.99	3.78	3.60	3.41	3.22	3.03	2.88	2.73	2.61	2.53	2.46	2.39	2.35	2.31	2.28	2.26	2.25	2.25
Upper 95% CI	5.74	5.33	4.98	4.69	4.45	4.23	4.03	3.85	3.68	3.47	3.30	3.13	2.98	2.88	2.78	2.70	2.63	2.58	2.54	2.54	2.60	2.71	2.87
Lower 95% CI	4.75	4.60	4.42	4.21	3.98	3.75	3.53	3.34	3.15	2.96	2.77	2.62	2.48	2.35	2.28	2.21	2.16	2.12	2.08	2.02	1.92	1.78	1.62

(D) Eastern Europe

Year	2001	2002	2003	2004	2005	2006	2007	2008	2009	2010	2011	2012	2013	2014	2015	2016	2017	2018	2019	2020	2021	2022	2023
LOESS smoothed rate	5.98	5.77	5.54	5.29	5.03	4.76	4.48	4.16	3.88	3.61	3.38	3.24	3.14	3.06	3.03	3.00	2.98	2.97	2.98	2.99	3.02	3.07	3.12
Upper 95% CI	6.84	6.41	6.04	5.72	5.45	5.19	4.91	4.60	4.32	4.03	3.80	3.64	3.55	3.45	3.40	3.37	3.34	3.32	3.32	3.35	3.45	3.63	3.87
Lower 95% CI	5.13	5.13	5.04	4.86	4.61	4.33	4.04	3.72	3.43	3.20	2.96	2.84	2.73	2.68	2.66	2.63	2.61	2.62	2.63	2.63	2.60	2.51	2.38

(E) Asia

Year	2001	2002	2003	2004	2005	2006	2007	2008	2009	2010	2011	2012	2013	2014	2015	2016	2017	2018	2019	2020	2021	2022	2023
LOESS smoothed rate	0.74	0.71	0.69	0.68	0.68	0.68	0.69	0.71	0.73	0.76	0.80	0.86	0.92	0.98	1.03	1.05	1.05	1.03	1.00	0.94	0.87	0.79	0.68
Upper 95% CI	1.18	1.03	0.94	0.90	0.90	0.91	0.92	0.93	0.96	0.99	1.05	1.10	1.18	1.23	1.26	1.28	1.27	1.25	1.23	1.25	1.30	1.38	1.48
Lower 95% CI	0.29	0.39	0.44	0.46	0.46	0.45	0.46	0.48	0.49	0.53	0.55	0.61	0.66	0.74	0.79	0.82	0.83	0.82	0.76	0.64	0.45	0.19	-0.12

(F) Oceania

Year	2001	2002	2003	2004	2005	2006	2007	2008	2009	2010	2011	2012	2013	2014	2015	2016	2017	2018	2019	2020	2021	2022	2023
LOESS smoothed rate	1.79	1.72	1.66	1.60	1.54	1.49	1.44	1.38	1.34	1.33	1.32	1.31	1.31	1.31	1.32	1.33	1.33	1.33	1.32	1.30	1.29	1.26	1.24
Upper 95% CI	2.15	1.99	1.87	1.79	1.74	1.69	1.64	1.58	1.55	1.52	1.52	1.50	1.50	1.51	1.50	1.50	1.50	1.49	1.48	1.47	1.48	1.51	1.57
Lower 95% CI	1.43	1.46	1.45	1.41	1.35	1.28	1.23	1.18	1.14	1.13	1.12	1.12	1.12	1.12	1.14	1.15	1.15	1.16	1.15	1.14	1.09	1.02	0.91

(G) Africa

Year	2001	2002	2003	2004	2005	2006	2007	2008	2009	2010	2011	2012	2013	2014	2015	2016	2017	2018	2019	2020	2021	2022	2023
LOESS smoothed rate	4.23	4.29	4.33	4.36	4.37	4.37	4.35	4.31	4.27	4.22	4.14	4.07	4.04	4.03	4.08	4.14	4.21	4.24	4.25	4.22	4.15	4.04	3.90
Upper 95% CI	4.64	4.59	4.57	4.56	4.58	4.58	4.57	4.54	4.50	4.45	4.37	4.31	4.27	4.27	4.31	4.37	4.43	4.46	4.51	4.59	4.71	4.84	5.00
Lower 95% CI	3.82	3.98	4.09	4.15	4.17	4.15	4.13	4.09	4.04	3.99	3.91	3.82	3.81	3.79	3.84	3.91	3.99	4.02	3.99	3.84	3.59	3.24	2.81

Data are number of deaths per 100,000 people. LOESS=locally weighted regression. CI=confidence interval.

Supplementary Table 9. Geographical breakdown list of countries included in LOESS analysis

(A) Northern America	Canada and United States of America	2
(B) Latin America and The Caribbean	Antigua and Barbuda, Argentina, Bahamas, Belize, Brazil, Chile, Colombia, Costa Rica, Cuba, Dominica, Ecuador, Grenada, Guatemala, Guyana, Jamaica, Mexico, Nicaragua, Panama, Paraguay, Saint Kitts and Nevis, Saint Lucia, Saint Vincent and Grenadines, Suriname, Uruguay, and Venezuela	25
(C) Western Europe	Austria, Belgium, Denmark, Finland, France, Germany, Iceland, Ireland, Italy, Luxembourg, Malta, Netherlands, Norway, Portugal, Spain, Sweden, Switzerland, and United Kingdom	18
(D) Eastern Europe	Croatia, Cyprus, Czech Republic, Estonia, Hungary, Israel, Latvia, Lithuania, Poland, Republic of Moldova, Romania, Serbia, Slovakia, Slovenia, and Turkey	15
(E) Asia	Armenia, Georgia, Japan, Kuwait, Kyrgyzsatn, Philippines, Republic of Korea, Thailand, and Uzbekistan	10
(F) Oceania	Australia and New Zealand	2
(G) Africa	Mauritius and South Africa	2
Total		73

LOESS=locally weighted regression.

Supplementary Table 10. Age-standardised pulmonary embolism-related mortality rates for 73 countries by income level

(A) High Income

Year	2001	2002	2003	2004	2005	2006	2007	2008	2009	2010	2011	2012	2013	2014	2015	2016	2017	2018	2019	2020	2021	2022	2023
LOESS smoothed rate	3.68	3.52	3.37	3.22	3.08	2.95	2.82	2.70	2.59	2.48	2.39	2.31	2.23	2.17	2.11	2.07	2.05	2.04	2.05	2.06	2.09	2.14	2.20
Upper 95% CI	4.08	3.82	3.60	3.42	3.28	3.16	3.03	2.92	2.82	2.70	2.62	2.53	2.46	2.38	2.32	2.28	2.25	2.24	2.24	2.28	2.38	2.52	2.71
Lower 95% CI	3.28	3.22	3.14	3.02	2.88	2.74	2.61	2.48	2.36	2.26	2.17	2.09	2.01	1.96	1.91	1.87	1.85	1.85	1.85	1.84	1.81	1.75	1.68

(B) Upper-middle Income

Year	2001	2002	2003	2004	2005	2006	2007	2008	2009	2010	2011	2012	2013	2014	2015	2016	2017	2018	2019	2020	2021	2022	2023
LOESS smoothed rate	3.60	3.47	3.35	3.24	3.15	3.08	3.02	2.97	2.93	2.91	2.90	2.93	2.95	2.97	2.99	2.99	2.98	2.96	2.93	2.89	2.84	2.78	2.71
Upper 95% CI	4.04	3.79	3.59	3.46	3.37	3.30	3.24	3.19	3.16	3.13	3.13	3.14	3.18	3.18	3.19	3.20	3.18	3.16	3.14	3.13	3.15	3.20	3.28
Lower 95% CI	3.16	3.15	3.10	3.03	2.94	2.85	2.79	2.74	2.70	2.69	2.67	2.71	2.73	2.75	2.78	2.78	2.78	2.77	2.73	2.65	2.53	2.36	2.14

(C) Lower-middle Income

Year	2001	2002	2003	2004	2005	2006	2007	2008	2009	2010	2011	2012	2013	2014	2015	2016	2017	2018	2019	2020	2021	2022	2023
LOESS smoothed rate	0.92	0.96	0.99	1.01	1.03	1.06	1.08	1.11	1.14	1.24	1.37	1.57	1.77	1.82	1.80	1.81	1.98	2.23	2.57	3.01	3.53	4.13	4.82
Upper 95% CI	1.81	1.61	1.50	1.47	1.48	1.52	1.54	1.56	1.58	1.69	1.86	2.05	2.27	2.30	2.24	2.24	2.38	2.63	3.05	3.66	4.45	5.41	6.52
Lower 95% CI	0.04	0.30	0.47	0.55	0.58	0.60	0.63	0.66	0.70	0.80	0.88	1.09	1.26	1.34	1.35	1.38	1.58	1.82	2.09	2.35	2.61	2.86	3.12

Data are number of deaths per 100,000 people. LOESS=locally weighted regression. CI=confidence interval.

Supplementary Table 11.Income level breakdown list of countries included in LOESS analysis

(A) High Income	Australia, Austria, Bahamas, Belgium, Canada, Croatia, Cyprus, Czech Republic, Denmark, Estonia, Finland, France, Germany, Hungary, Iceland, Ireland, Israel, Italy, Japan, Kuwait, Luxembourg, Malta, Netherlands, New Zealand, Norway, Poland, Portugal, Republic of Korea, Saint Kitts and Nevis, Slovakia, Slovenia, Spain, Sweden, Switzerland, United Kingdom, and United States of America	36
(B) Upper-middle Income	Antigua and Barbuda, Argentina, Brazil, Chile, Colombia, Costa Rica, Cuba, Dominica, Ecuador, Grenada, Jamaica, Latvia, Lithuania, Mauritius, Mexico, Panama, Romania, Saint Lucia, Saint Vincent and Grenadines, Serbia, South Africa, Suriname, Thailand, Turkey, Uruguay, and Venezuela	26
(C) Lower-middle Income	Armenia, Belize, Georgia, Guatemala, Guyana, Nicaragua, Paraguay, Philippines, Republic of Moldova, and Uzbekistan	10
Total		72

*Low income country (only Kyrgyzstan) was not included in income level analysis. LOESS=locally weighted regression.

Supplementary Table 12. Average annual per cent changes in age-standardised pulmonary embolism-related mortality rate.

Country	Gender	Period	AAPC (%) [95% CI]	P-Value
Australia	Both	2010-2023	-0.8 [-1.9 , 0.3]	0.16
Austria	Both	2010-2023	-3.9 * [-5.3 , -2.4]	p<0.0001
Czech Republic	Both	2010-2023	-5.7 * [-7.2 , -3.7]	p<0.0001
Hungary	Both	2010-2023	-3.4 * [-5.1 , -1.7]	0.0004
Latvia	Both	2010-2023	3.6 * [1.9 , 7.2]	p<0.0001
Lithuania	Both	2010-2023	-1.3 [-2.6 , 0.6]	0.15
Mauritius	Both	2010-2023	-2.9 [-6.6 , 2.1]	0.23
Netherlands	Both	2010-2023	-2.7 * [-4.1 , -1.6]	p<0.0001
Serbia	Both	2010-2023	0.8 [-0.6 , 2.3]	0.22
Sweden	Both	2010-2023	-1.7 * [-2.7 , -0.6]	0.0044
Turkey	Both	2010-2023	0.8 [-0.6 , 2.4]	0.29
Argentina	Both	2010-2022	0.5 [-0.2 , 1.4]	0.16
Armenia	Both	2010-2022	-0.8 [-3.6 , 1.7]	0.51
Canada	Both	2010-2022	-0.2 [-1.2 , 1.2]	0.85
Costa Rica	Both	2010-2022	-3.7 * [-6.4 , -1.7]	0.0020
Cyprus	Both	2010-2022	3.3 [-1.1 , 8.6]	0.12
Denmark	Both	2010-2022	-0.9 [-3.0 , 0.5]	0.16
Ecuador	Both	2010-2022	-3.1 * [-5.2 , -0.7]	0.0060
Estonia	Both	2010-2022	2.7 [-0.2 , 6.1]	0.062
Finland	Both	2010-2022	-3.8 * [-5.3 , -2.4]	p<0.0001
France	Both	2010-2022	-4.3 * [-4.9 , -3.5]	p<0.0001
Georgia	Both	2010-2023	9.4 * [4.0 , 22.7]	0.0008
Germany	Both	2010-2022	-5.0 * [-5.9 , -4.2]	p<0.0001
Guatemala	Both	2010-2022	2.6 [-0.5 , 4.6]	0.10
Iceland	Both	2010-2022	-1.0 [-3.3 , 1.0]	0.28
Ireland	Both	2010-2022	-1.5 * [-3.6 , -0.2]	0.027
Israel	Both	2010-2022	-2.6 * [-4.3 , -0.8]	0.0056
Luxembourg	Both	2010-2022	-8.6 * [-11.6 , -5.5]	p<0.0001
Mexico	Both	2010-2022	1.9 * [0.7 , 3.3]	0.0020
Nicaragua	Both	2010-2022	28.4 * [16.8 , 65.8]	p<0.0001
Poland	Both	2010-2022	-2.9 * [-3.7 , -2.2]	p<0.0001
Republic of Korea	Both	2010-2022	0.5 [-0.6 , 1.8]	0.32
Spain	Both	2010-2022	0.9 * [0.3 , 1.4]	0.0048
Switzerland	Both	2010-2022	-0.8 * [-1.5 , -0.1]	0.038
United States of America	Both	2010-2022	0.6 * [0.1 , 1.1]	0.018
Antigua and Barbuda	Both	2010-2021	-2.8 [-13.4 , 8.2]	0.51
Belgium	Both	2010-2021	-3.6 * [-5.0 , -2.3]	p<0.0001
Brazil	Both	2010-2021	-1.7 [-3.4 , 0.1]	0.064
Chile	Both	2010-2021	4.3 * [2.4 , 6.4]	p<0.0001
Colombia	Both	2010-2021	-1.6 [-5.0 , 0.4]	0.096
Croatia	Both	2010-2021	-6.8 * [-12.2 , -2.4]	0.0012
Cuba	Both	2010-2021	15.5 * [11.6 , 19.9]	p<0.0001
Grenada	Both	2010-2021	0.7 [-10.1 , 12.0]	0.84
Italy	Both	2010-2021	-0.8 [-1.9 , 0.3]	0.15
Japan	Both	2010-2021	-3.3 * [-5.1 , -1.6]	p<0.0001
Kuwait	Both	2010-2023	-9.5 * [-16.0 , -3.7]	0.0012
Malta	Both	2010-2021	3.1 [-2.4 , 9.9]	0.24
Panama	Both	2010-2022	-4.8 * [-6.8 , -2.9]	p<0.0001
Paraguay	Both	2010-2021	-1.9 [-4.4 , 1.2]	0.23
Saint Vincent and Grenadines	Both	2010-2021	4.4 [-3.5 , 14.2]	0.21
Slovakia	Both	2010-2023	-0.9 [-4.0 , 2.5]	0.57
United Kingdom	Both	2010-2021	-2.9 * [-3.9 , -1.4]	p<0.0001
Uruguay	Both	2010-2022	-2.8 [-4.8 , 0.2]	0.064
Dominica	Both	2010-2020	-13.3 [-41.2 , 13.2]	0.17

Portugal	Both	2010-2022	-1.0	[-4.8 , 2.7]	0.52
Saint Lucia	Both	2010-2020	-6.2	[-12.1 , 0.2]	0.059
Singapore	Both	2012-2022	-3.7 *	[-6.6 , -0.7]	0.012
Slovenia	Both	2010-2020	-6.8 *	[-8.3 , -5.6]	p<0.0001
South Africa	Both	2010-2020	0.2	[-0.4 , 1.1]	0.40
Thailand	Both	2010-2021	7.4 *	[5.7 , 10.1]	p<0.0001
Bosnia and Herzegovina	Both	2011-2023	3.3 *	[0.5 , 6.8]	0.021
Guyana	Both	2010-2019	-4.5	[-13.4 , 7.4]	0.31
Kazakhstan	Both	2013-2022	5.8	[-3.0 , 17.6]	0.18
Kyrgyzstan	Both	2010-2019	3.9 *	[1.0 , 7.2]	0.0088
Republic of Moldova	Both	2010-2021	4.5 *	[1.6 , 8.0]	0.0004
Romania	Both	2010-2019	2.9 *	[0.1 , 7.1]	0.036
Uzbekistan	Both	2010-2019	18.0 *	[12.9 , 28.1]	p<0.0001
Brunei Darussalam	Both	2011-2019	5.5	[-3.6 , 24.1]	0.19
New Zealand	Both	2010-2018	6.5 *	[1.8 , 12.2]	0.011
Greece	Both	2014-2021	-1.6	[-4.1 , 0.1]	0.063
Mongolia	Both	2016-2023	5.0	[-3.1 , 14.9]	0.20
Belize	Both	2010-2016	-4.7	[-10.3 , 1.0]	0.10
Norway	Both	2010-2016	-1.5	[-5.1 , 2.2]	0.43
Philippines	Both	2010-2019	-2.4	[-5.7 , 1.2]	0.18
Venezuela	Both	2010-2016	6.2 *	[2.6 , 10.8]	p<0.0001

* Indicates that average annual per cent changes (AAPCs) were statistically significantly different from zero (p<0.05)
